# Black-Box Simulations of Anharmonic Vibrational Chiroptical Spectra: Problems with Property Third Derivatives and the Solvent

**DOI:** 10.1021/acs.jctc.5c01132

**Published:** 2025-10-13

**Authors:** Qin Yang, Valery Andrushchenko, Jana Hudecová, Josef Kapitán, Julien Bloino, Isabelle Bowker, Petr Bouř

**Affiliations:** † Institute of Organic Chemistry and Biochemistry, Academy of Sciences, Flemingovo náměstí 2, Prague 16610, Czech Republic; ‡ Department of Optics, 48207 Palacký University Olomouc, 17. listopadu 12, Olomouc 77900, Czech Republic; # 19004 Scuola Normale Superiore di Pisa, Piazza dei Cavalieri 7, Pisa 56126, Italy

## Abstract

Chiroptical methods, including vibrational circular dichroism (VCD) and Raman optical activity (ROA), reveal details about molecular structure. For three model molecules, α-pinene, camphor, and fenchone, we show that increased sensitivity of modern spectrometers makes it possible to record even fine spectral features, such as overtone and combination bands. However, understanding, interpretation, and simulation of them require relatively expensive computations, going beyond the harmonic approximation. For this purpose, vibrational perturbation theory at the second order (VPT2) has proven to provide an excellent price-performance balance. As it becomes more common, inconsistencies in electronic structure calculations, hidden by error compensation at the harmonic level, emerge. In particular, while trying to interpret the spectra, we found that the commonly used polarizable continuum models (PCM) of solvent may introduce erroneous perturbations to the higher derivatives of dipole moments and polarizabilities needed to simulate spectral intensities. We therefore analyze the experimental spectra on the basis of the simulations and explore parameters allowing for a “black-box” VPT2 application. In particular, explicit cavities used for the hydrogen atoms resulted in excessively large third derivatives of molecular polarizabilities and sometimes led to incorrect signs of ROA and VCD bands, even for fundamental transitions. This could be partially rectified by a combination of different approximation levels used for the calculation of different properties, or by using PCM cavities not explicitly adapted for hydrogen atoms. Under these conditions, VPT2 combined with a proper treatment of resonances appears as an excellent tool to simulate and understand the spectra, including the assignment of weak anharmonic bands.

## Graphical abstract

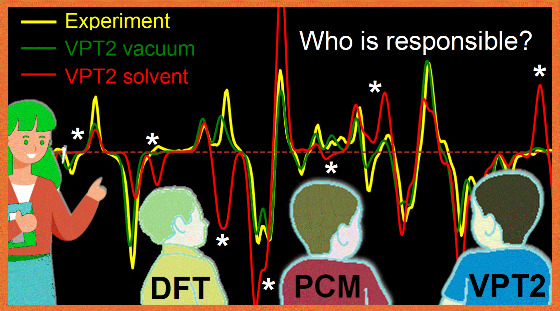

## Introduction

The spectroscopy of vibrational optical activity (VOA) has been established as an excellent choice to study molecular structure in many fields of biochemistry, organic and physical chemistry, or material science.^1–6^ It comprises two main branches: vibrational circular dichroism (VCD) and Raman optical activity (ROA). Compared to unpolarized spectroscopies (IR, Raman), VOA spectral bands can be both positive and negative, thus enabling better resolution of individual vibrations and carrying additional information about the dynamics and geometry of the studied systems.

However, anharmonic features in the vibrational spectra, typically overtone and combination bands, still constitute relatively unknown territories that VOA spectroscopy is starting to explore. The recording of such bands is possible due to the increased sensitivity of modern spectrometers. The intensities and positions of the ″anharmonic″ peaks reveal vibrational mode coupling and further details of the molecular potential energy and property surfaces.^7–9^ However, their interpretation based on ab initio modeling requires significantly greater computational effort than analyses of fundamental vibrational transitions.

For the description of the electronic wave function in larger molecules, density functional theory (DFT) currently appears as a reasonable possibility, giving a good balance of accuracy and computational cost.^10^ Similarly, polarizable continuum models (PCM) of solvent allow one to describe the solvent and environmental effects in a computationally convenient way,^11,12^ although they may account for hydrogen bonding and other strong interactions only partially.^13^ The models were nevertheless found fit for the spectral simulations, including anharmonic features.^14^

Methods suitable for the treatment of anharmonic vibrational properties include vibrational configuration interaction,^15^ vibrational self-consistent field,^16^ vibrational coupled cluster,^17^ and vibrational perturbation theory at the second order (VPT2).^18^ The last one is used in the present study as it provides several advantages, such as the availability of analytic formulas to compute the energies and intensities of VCD and ROA spectra. Compared to the variational alternatives, VPT2 requires a significantly shorter computational time and a smaller number of anharmonic constants necessary to reach convergence. It is usually able to recover the leading anharmonic contributions when applied to rigid and semirigid systems, providing sufficient accuracy for spectral interpretation.

It is true that traditional VPT2 formulas diverge at resonances. However, efficient countermeasures have been proposed, such as resonance identification based on energies or contributions to the intensities, and a correction through a limited variational treatment.^7,8,19–27^ Still, there are a limited number of tests to confirm that existing protocols can be used in a black-box way. Thus, when applied to larger molecules (say, of more than 30 atoms), the resonance pattern becomes complicated and needs to be treated carefully.^19,22^ Nevertheless, recent results obtained by some of us have shown that the so-called generalized VPT2 (GVPT2) scheme can provide accurate results on par with experiment.^7,9,28^ In any case, the outcome of the spectral simulations in individual cases is difficult to predict, as they involve many steps, such as modeling of the electronic structure, representation of the environmental effects, or treatment of conformational equilibria.

With this in mind, we chose three molecules to assess the performance of the computational apparatus and identify possible problems. α-Pinene, camphor, and fenchone (Figure 1) are convenient models for many reasons. They are relatively small, so they can be extensively studied even with relatively expensive theoretical models; their structure is rigid, so that conformer modeling issues are minimized, and they exhibit large VOA signals.^7^ The signal strength made it possible to measure some combination and overtone bands. Rather surprisingly, when comparing the experimental and simulated spectra, we found that GVPT2 did not always provide reliable results, which is at odds with previous studies.

**Figure 1:**
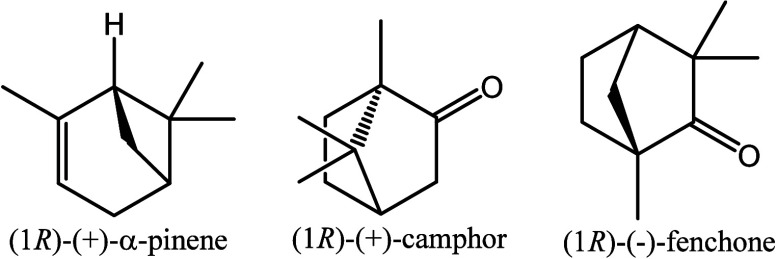
Structures of the reference compounds.

A deeper analysis showed that it was the application of PCM that was problematic. Its usage in numerical differentiation was questioned before for energy derivatives, nevertheless, without a detailed analysis.^29^ We did not find problems with frequencies; however, it appeared that PCM could distort third derivatives of molecular polarizabilities, contributing to anharmonic features in the spectra. These errors are relatively minor for nonpolar compounds and might have been overlooked in the past,^14^ but become more serious for compounds containing polar groups. Fortunately, as shown below, a protocol suitable for simulations in an automatic way can be devised, including the solvent. We thus believe that our findings will be useful to researchers interested in the interpretations of vibrational experiments, be it in analytical, biochemical, or other VOA applications.

## Methodology

### Spectra Measurement

All chemicals were obtained from Sigma-Aldrich. For IR and VCD, both enantiomers of camphor were dissolved in deuterated chloroform (CDCl_3_) and placed in a sealed hexagonal BaF_2_ cell (ICL, Inc., Garfield, USA) with 100 μm path length (200 μm for the 2800–1800 cm^–1^ and 4550–3100 cm^–1^ regions). The concentration was 225 mg/mL (for the region 1800–830 cm^–1^), 60 mg/mL (for 3100–2700 cm^–1^), and saturated solution (∼ 5 M, for the other ″anharmonic″ regions). CCl_4_ was used as a solvent instead of CDCl_3_ for ν­(C = O) measurements. Fenchone and α-pinene enantiomers were measured as neat compounds, except for α-pinene in the 3100–2700 cm^–1^ region, where 0.252 M solution in CDCl_3_ was used. Within 1570–750 cm^–1^, a dismountable cell with round BaF_2_ windows (ICL, Inc., Garfield, USA) and 25 μm spacer were used; for the 3100–2700 cm^–1^ region, a custom-made dismountable CaF_2_ cell with a path length of 6 μm was used for fenchone, while the sealed hexagonal BaF_2_ cell with 100 μm path length was used for α-pinene. For dismountable cells, 20–50 μL was placed on the bottom window, covered with the top one, and the cell was tightened. A sealed hexagonal BaF_2_ cell with 200 μm path length was used for the other regions. The spectra were recorded with a ChiralIR-2X VCD spectrometer (BioTools, Inc., Jupiter, USA) with a resolution of 4 cm^–1^ at room temperature. The accumulation times were several hours for the fingerprint region and up to 20 h for the others, to achieve a sufficient signal-to-noise ratio. Spectra of the solvent, empty cell windows, or a racemic mixture of both enantiomers were measured under the same conditions as the samples and subtracted as a baseline; the resulting spectra were then subjected to additional minor baseline correction and expressed in standard units (Δε and ε, in L·cm^–1^·mol^–1^).

Raman and ROA spectra were measured on a Zebr spectrometer^8^ at 20 °C, within 50 to 4550 cm^–1^, using 532 nm excitation wavelength, the backscattering geometry, and scattered circular polarization (SCP) modulation scheme. The samples were held in a rectangular fused silica cell (70 μL volume). Laser powers and accumulation times are given in Table S1. About 10 times longer times were needed to accumulate the weak signal of the overtone and combination bands than for the fundamentals. The intensities are presented in cm·J^–1^ (electron count per wavenumber interval per irradiation energy). α-Pinene and fenchone were measured as neat liquids. Camphor was dissolved at a concentration of 500 mg/mL in CHCl_3_ or MeOH. The solvent signal could not be fully subtracted from the camphor spectra, in particular CHCl_3_ bands at 259, 364, 666, 758, 2399 cm^–1^, and the 3343 cm^–1^ band of MeOH (Figure S1). A minor baseline correction was applied to the ROA spectra.

### GVPT2 Computations

The Gaussian suite of quantum chemical programs^30^ and its development version^31^ were used for the DFT and vibrational calculations. The B3PW91 ^32^/jun-cc-pVTZ combination was chosen as a reference approximation level, having given reasonable results in previous studies.^22^ The 6–311++G­(d,p) basis set provided nearly the same results as jun-cc-pVTZ, and was used for some tests to save computational time. Other functionals tested included HF, B2PLYP,^33^ Cam-B3LYP,^34^ and B3LYP.^35^ An empirical dispersion correction^36^ was used throughout. Solvent and environmental effects were included by means of the PCM model;^12^ methylcyclohexane parameters were used to mimic neat pinene and fenchone. The solvent enters expressions for optical activity tensors through the molecular Hamiltonian; details about the implementation of chiral properties within the PCM framework can be found in refs 37 and 38. For tests, dielectric constants, atomic radii, and other PCM parameters were varied.

The VPT2 vibrational potential was

$$V=\frac{1}{2}\underset{i}{\sum }\omega_{i}q_{i}^{2}+\frac{1}{6}\underset{i,j,k}{\sum }f_{ijk}q_{i}q_{j}q_{k}+\frac{1}{24}\underset{i,j,k,l}{\sum }f_{ijkl}q_{i}q_{j}q_{k}q_{l}$$ 1

where *V*, ω_*i*_, *f*_*ijk*_, and *f*_*ijkl*_ had units of cm^–1^, ω_*i*_ is a fundamental frequency, *q*_*i*_ is a reduced normal-mode coordinate, *f*_*ijk*_ */f*_*ijkl*_ are the third/fourth energy derivatives. The anharmonic constants (all *f*_*ijk*_, semidiagonal *f*_*ijkl*_) were calculated by a two-step numerical differentiation in the normal modes, a default differentiation step along the mass-weighted normal coordinates *Q*_*i*_ was 0.01 amu^1/2^ Å. Coriolis couplings were also included in the second-order correction of the Hamiltonian, as the zeroth-order term with respect to rotational quanta from Watson’s rovibrational Hamiltonian. These, however, do not significantly contribute to spectral intensities.^7^

Similarly, the electric μ and magnetic *m* dipole moments (needed for IR and VCD), electric α, magnetic *G*′, and quadrupole A polarizabilities (for Raman and ROA)^39^ are expanded as

$$\mu_{\alpha }=\mu_{0,\alpha }+\underset{i}{\sum }P_{\alpha i}q_{i}+\frac{1}{2}\underset{i,j}{\sum }\frac{\partial P_{\alpha i}}{\partial q_{j}}q_{i}q_{j}+\frac{1}{6}\underset{i,j,k}{\sum }\frac{\partial^{2}P_{\alpha i}}{\partial q_{j}\partial q_{k}}q_{i}q_{j}q_{k}$$ 2a

$$m_{\alpha }=m_{0,\alpha }+\underset{i}{\sum }A_{\alpha i}p_{i}+\underset{i,j}{\sum }\frac{\partial A_{\alpha i}}{\partial q_{j}}p_{i}q_{j}+\frac{1}{2}\underset{i,j,k}{\sum }\frac{\partial^{2}A_{\alpha i}}{\partial q_{j}\partial q_{k}}p_{i}q_{j}q_{k}$$ 2b

$$\alpha_{\alpha \beta }=\alpha_{0,\alpha \beta }+\underset{i}{\sum }\frac{\partial \alpha_{\alpha \beta }}{\partial q_{i}}q_{i}+\frac{1}{2}\underset{i,j}{\sum }\frac{\partial^{2}\alpha_{\alpha \beta }}{\partial q_{i}\partial q_{j}}q_{i}q_{j}+\frac{1}{6}\underset{i,j,k}{\sum }\frac{\partial^{3}\alpha_{\alpha \beta }}{\partial q_{i}\partial q_{j}\partial q_{k}}q_{i}q_{j}q_{k}$$ 2c

$${G^{\prime }}_{\alpha \beta }={G^{\prime }}_{0,\alpha \beta }+\underset{i}{\sum }\frac{{\partial G^{\prime }}_{\alpha \beta }}{\partial q_{i}}q_{i}+\frac{1}{2}\underset{i,j}{\sum }\frac{\partial^{2}{G^{\prime }}_{\alpha \beta }}{\partial q_{i}\partial q_{j}}q_{i}q_{j}+\frac{1}{6}\underset{i,j,k}{\sum }\frac{\partial^{3}{G^{\prime }}_{\alpha \beta }}{\partial q_{i}\partial q_{j}\partial q_{k}}q_{i}q_{j}q_{k}$$ 2d

$$A_{\alpha ,\beta \gamma }=A_{0,\alpha ,\beta \gamma }+\underset{i}{\sum }\frac{\partial A_{\alpha ,\beta \gamma }}{\partial q_{i}}q_{i}+\frac{1}{2}\underset{i,j}{\sum }\frac{\partial^{2}A_{\alpha ,\beta \gamma }}{\partial q_{i}\partial q_{j}}q_{i}q_{j}+\frac{1}{6}\underset{i,j,k}{\sum }\frac{\partial^{3}A_{\alpha ,\beta \gamma }}{\partial q_{i}\partial q_{j}\partial q_{k}}q_{i}q_{j}q_{k}$$ 2e

where the derivatives are taken at the equilibrium positions, $P_{\alpha i}=\frac{\partial \mu_{\alpha }}{\partial q_{j}}$ and $A_{\alpha i}=\frac{\partial m_{\alpha }}{\partial p_{j}}$ are the atomic polar and axial tensors, respectively, transformed to the normal-mode coordinates, *p*_*i*_ is the momentum related to the *q*_*i*_ coordinate. As *P*_α*i*_, *A*_α*i*_, $\frac{\partial \alpha_{\alpha \beta }}{\partial q_{i}}$ , $\frac{{\partial G^{\prime }}_{\alpha \beta }}{\partial q_{i}}$ , and $\frac{\partial A_{\alpha ,\beta \gamma }}{\partial q_{i}}$ are obtained analytically from the Gaussian program, the same two-point differentiation as used for higher-energy derivatives provided all components of $\frac{\partial P_{\alpha i}}{\partial q_{j}}$ , $\frac{\partial A_{\alpha i}}{\partial q_{j}}$ , $\frac{\partial^{2}\alpha_{\alpha \beta }}{\partial q_{i}\partial q_{j}}$ , $\frac{\partial^{2}{G^{\prime }}_{\alpha \beta }}{\partial q_{i}\partial q_{j}}$ , and $\frac{\partial^{2}A_{\alpha ,\beta \gamma }}{\partial q_{i}\partial q_{j}}$ as well as the semidiagonal third derivatives of μ, *m*, α, *G*′, and *A*. The anharmonicity stemming from the potential (eq 1) and its wave function is commonly referred to as the mechanical anharmonicity. For simplicity, second and higher derivatives of the electromagnetic tensors (in eqs 2a–2e) will be further called electrical or property anharmonicity.

VPT2 wave functions ψ are not normalized, and a transition moment of a generic property *X* is calculated as

$${\left\langle X\right\rangle }_{I,F}=\frac{\left\langle \psi_{F}\right|X\left|\psi_{I}\right\rangle }{\sqrt{\left\langle \psi_{F}\right|\psi_{F}\rangle \left\langle \psi_{I}\right|\psi_{I}\rangle }}$$ 3

For a deeper view into how the wave function and electric anharmonicities mix, we can divide *X* in the harmonic (*X* ^(0)^, first two terms in eqs 2a–2e), first and second (*X* ^(1)^ and *X* ^(^^2)^, third and fourth terms in eqs 2a–2e) order contributions. Then, eq 3 develops into

$$\begin{array}{l} {\left\langle X\right\rangle }_{I,F}=\left\langle \psi_{F}^{\left(0\right)}\right|X^{\left(0\right)}{\left|\psi_{I}^{\left(0\right)}\right\rangle }_{1}\\ +\left\langle \psi_{F}^{\left(0\right)}\right|X^{\left(1\right)}{\left|\psi_{I}^{\left(0\right)}\right\rangle }_{2}+\left\langle \psi_{F}^{\left(1\right)}\right|X^{\left(0\right)}{\left|\psi_{I}^{\left(0\right)}\right\rangle }_{3}+\left\langle \psi_{F}^{\left(0\right)}\right|X^{\left(0\right)}{\left|\psi_{I}^{\left(1\right)}\right\rangle }_{4}\\ +\left\langle \psi_{F}^{\left(0\right)}\right|X^{\left(2\right)}{\left|\psi_{I}^{\left(0\right)}\right\rangle }_{5}+\left\langle \psi_{F}^{\left(2\right)}\right|X^{\left(0\right)}{\left|\psi_{I}^{\left(0\right)}\right\rangle }_{6}+\left\langle \psi_{F}^{\left(0\right)}\right|X^{\left(0\right)}{\left|\psi_{I}^{\left(2\right)}\right\rangle }_{7}\\ +\left\langle \psi_{F}^{\left(1\right)}\right|X^{\left(1\right)}{\left|\psi_{I}^{\left(0\right)}\right\rangle }_{8}+\left\langle \psi_{F}^{\left(0\right)}\right|X^{\left(1\right)}{\left|\psi_{I}^{\left(1\right)}\right\rangle }_{9}+\left\langle \psi_{F}^{\left(1\right)}\right|X^{\left(0\right)}{\left|\psi_{I}^{\left(1\right)}\right\rangle }_{10}\\ -\frac{1}{2}\left\langle \psi_{F}^{\left(0\right)}\right|X^{\left(0\right)}\left|\psi_{I}^{\left(0\right)}\right\rangle {\left(\left\langle \psi_{F}^{\left(1\right)}\right|\psi_{F}^{\left(1\right)}\rangle +\left\langle \psi_{I}^{\left(1\right)}\right|\psi_{I}^{\left(1\right)}\rangle \right)}_{11} \end{array}$$ 4

where we added indices for easier identification. The first term corresponds to the harmonic-oscillator approximation. Terms 3, 4, 6, 7, 10, and 11 with *X* ^(0)^ depend on the harmonic components of the property; the so-called mixed anharmonic terms 8 and 9, with *X* ^(1)^, contain both the anharmonicity of the wave function and property second derivatives. Terms number 2 and 5, containing the second *X* ^(1)^ and third *X* ^(2)^ derivatives, respectively, represent a “pure” electrical anharmonicity.

The treatment of resonances follows the scheme described in ref 28. In it and references therein, multiple criteria for resonance identification were developed. For Fermi resonances, three conditions are employed,

$$|\omega_{k}-(\omega_{i}+\omega_{j})|\leq 200\;{\mathrm{cm}}^{-1}$$ 5a

$$f_{ijk}^{4}\geq 64{\left(1+\delta_{jk}\right)}^{2}{\left|\omega_{k}-\left(\omega_{i}+\omega_{j}\right)\right|}^{2}$$ 5b

$$\left|f_{ijk}\right|\geq 0.02\left|\left(\omega_{i}-\omega_{k}+\omega_{j}\right)\sqrt{2\left(1+\delta_{jk}\right)}\right|$$ 5c

For Darling–Dennison resonances (1–1, between fundamentals and 2–2 between overtones and 1 + 1 combinations), three (6a–c, for 1–1) or two (6a–b, for 2–2) conditions were evaluated,

$$|\omega_{i}-\omega_{j}|\;\mathrm{or}\;|\omega_{i}+\omega_{j}-\omega_{k}-\omega_{l}|\leq 200\;{\mathrm{cm}}^{-1}$$ 6a

$$|\langle 1_{i}|\mathrm{H̃}|1_{j}\rangle |\;\mathrm{or}\;|\langle 1_{i}1_{j}|\mathrm{H̃}|1_{k}1_{l}\rangle |\geq 10\;{\mathrm{cm}}^{-1}$$ 6b

$$\max\left(c_{1_{i}1_{j}}^{\left(2\right)},c_{1_{j}1_{i}}^{\left(2\right)}\right)\geq 0.03$$ 6c

where 1_*i*_ indicates one quantum in mode *i, H̃* is a contact-transformed Hamiltonian. For Fermi and 2–2 Darling–Dennison resonances, the indices are repeated for overtones (e.g., 1_i_1_i_). The criterion (6c) with the coefficients *c* of the VPT2 wave function is specific to the intensity and is used to identify potential singularities in the calculation of transition moments between weakly coupled states (*f*_*ijk*_ of small magnitude in the case of Fermi resonances). The resonant terms were removed from the VPT2 treatment and then introduced back through a variational step. The overall procedure is therefore termed generalized VPT2 (GVPT2).

From the calculated frequencies and intensities, smooth spectra were generated via convolution with a Lorentzian function, using 8 cm^–1^ of full width at half-maximum. Following the usual procedures,^40^ similarity factors *s* between computed and experimental spectra were calculated as scalar products of normalized spectra for the best scaling factor *a*, i.e., *s* = min [*s*(*a*)], *s*(*a*) = $\int S_{Cal}(a\omega )S_{\exp}(\omega )d\omega /$ $\sqrt{\int S_{Cal}^{2}(a\omega )d\omega \int S_{\exp}^{2}(\omega^{\prime })d\omega }$ , so that *s* = 1 indicates identical spectra. The scaling factors were very close to one, especially for the anharmonic simulations. An alternative comparison with unscaled spectra (*a* = 1) led to very similar results.

## Results

### Anharmonic Features in Experimental Spectra

ROA, Raman, VCD, and IR spectra of all three compounds are shown in Figure 2. Enantiomers are giving reasonable ″mirror images″ and the spectra are consistent with those obtained in previous studies.^8,25,41–45^ In addition, many anharmonic (combination and overtone) bands are visible, in particular, in ROA. These are typically about 1–2 orders of magnitude smaller than those from the fundamental transitions; however, also here, the mirror imaging of the enantiomeric spectra confirms that the signals are reliable. For α-pinene VCD, above 3000 cm^–1^, the very weak vibrational signal appears to be superimposed on broad baselines, likely caused by sample impurities (oxidation products of the terpene) or by instrumental artifacts. Another means to distinguish the desired vibrational signal from noise is provided by the simulations, as shown below. For camphor, such anharmonic ROA bands have not yet been reported for the solutions. In the figure, 104 spectral segments (4 spectral kinds × 3 compounds × 2 enantiomers × 4–5 wavenumber intervals) are plotted with different magnifications to be visible, which highlights one of the challenges in analyzing the anharmonic features.

**Figure 2:**
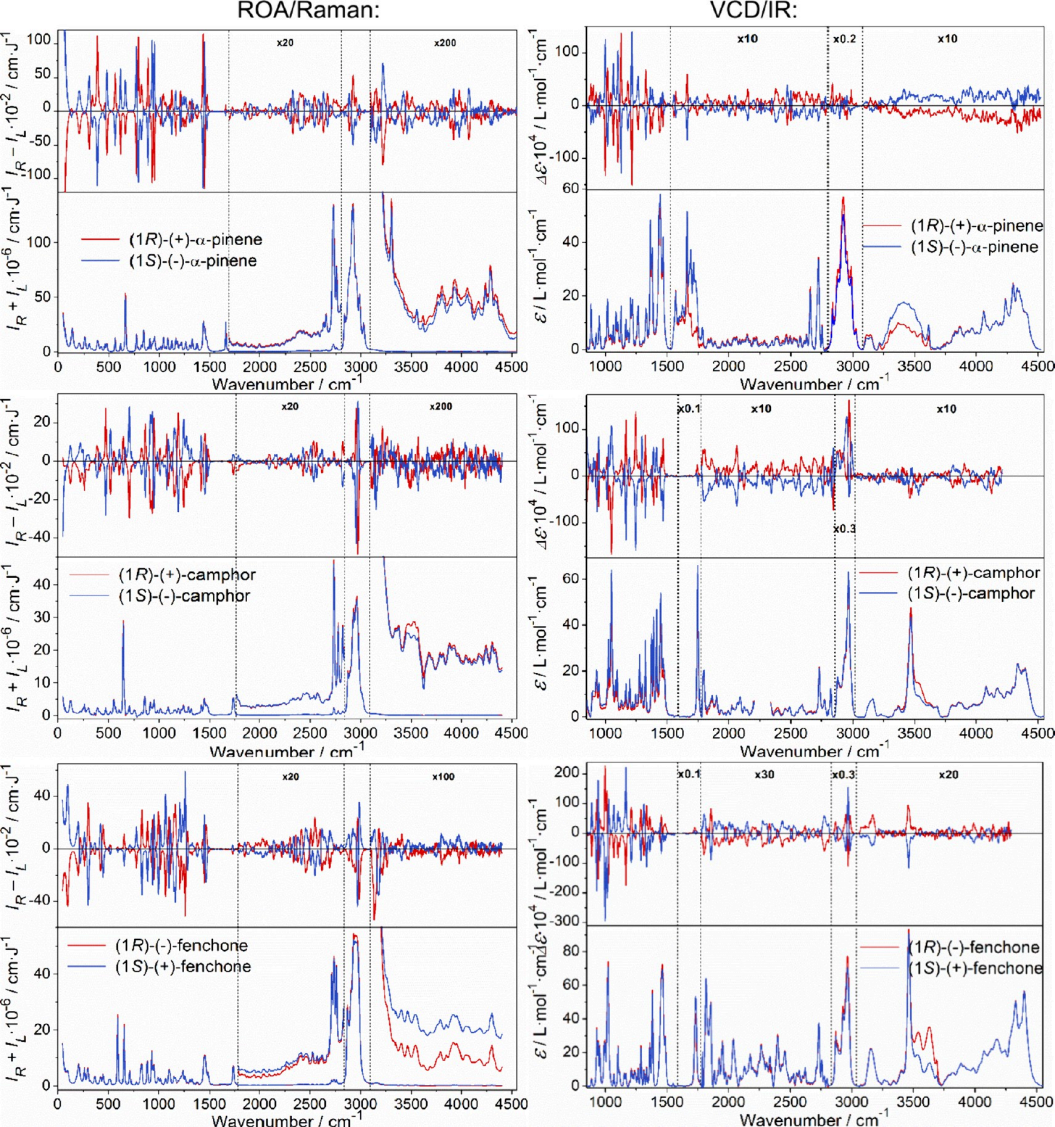
Experimental spectra of the three investigated molecules. Left column: ROA/Raman (*I*_*R*_ – *I*_*L*_/*I*_*R*_ *+ I*_*L*_), right column: VCD/absorption (Δε/ε). Intensities in some regions were multiplied as indicated.

For α-pinene, Raman and ROA spectra including the anharmonic features were reported before,^8^ albeit in a limited range (<3750 cm^–1^) and with a lower signal-to-noise ratio; the spectra in the present study are recorded up to 4500 cm^–1^, and the enantiomers give clearly opposite ROA, even for very tiny bands, more than 200-times weaker than the fundamentals. This demonstrates the fast improvement in today’s ROA technology and the need for the simulations to account for anharmonic contributions. The high-frequency region has been explored rather rarely,^25^ and in the first experiments, the anharmonic features were not visible at all.^44^ For camphor and fenchone, we are not aware of comparable measurements so far. For camphor, the ROA features above 3000 cm^–1^ were particularly difficult to accumulate since the compound had to be dissolved in chloroform or methanol. In the solutions, solvent and camphor bands may interfere, and the concentration is lower.

High-frequency IR and VCD spectra, including weak anharmonic bands, are explored more than Raman and ROA. For α-pinene and camphor, VCD bands even close to 9000 cm^–1^ were reported.^46^ A local-mode approximation was used to model the CH fundamental stretching bands of fenchone and camphor.^42^

### Problems with PCM Simulations

As shown before, the GVPT2 method can significantly improve the frequencies and VOA intensities obtained at the harmonic level.^7,9,28^ Accounting for the solvent using PCM in general further improves vibrational frequencies calculated in vacuum.^47^ For all compounds, in particular, for camphor and fenchone, however, we found that the GVPT2/PCM combination leads to unreasonable intensity of some simulated bands. In particular, the wrong ROA and VCD signs were predicted, and the overall agreement with the experiment was thus worse than for the harmonic calculation.

This is shown for (*R*)-camphor in Figure 3, where the optimal scaling factors and similarities to experiment are also indicated. For all the ROA, Raman, VCD, and IR spectra, the harmonic level qualitatively reproduces the experimental shapes, albeit most frequencies are shifted to higher values. Scaling factors close to 0.98 are needed for the best match below 1800 cm^–1^. The anharmonic (GVPT2) vacuum computation shows a clear improvement over the harmonic results, in particular, the C–O and C–H stretching frequencies. The default Gaussian^30^ PCM model^48^ and GVPT2 give even better frequencies than without PCM, in particular for the C–O stretching. However, the intensity pattern is significantly worse. For ROA, for example, about 19 bands are clearly predicted to have the wrong signs. The normal-mode analysis revealed that the biggest inconsistencies appear for vibrations involving a significant movement of the hydrogen atoms, such as C–H stretching and C–H bending motions (around 3000/1400 cm^–1^) while modes involving heavy atoms only (such as C–C stretching around 1100 cm^–1^) are affected less. This can be rectified when the united atom topological model (U, the last spectra in the sets) is used to construct the cavities. In this approach, heavy atoms and their bonded hydrogen atoms are grouped into single ″united″ particles, requiring only one spherical cavity.^30,48^ Such a combination provides reasonable wavenumbers and intensities. The optimal scaling factor is then slightly higher than one, indicating that the GVPT2 method slightly underestimates the transition energies. The average similarities are quite high, 0.84–0.91, although minor inconsistencies still exist, most likely explicable by a limited precision of the DFT and GPVT2 methods, neglect of the explicit solvent interactions, or approximations in the light-scattering theory.^49,50^

**Figure 3:**
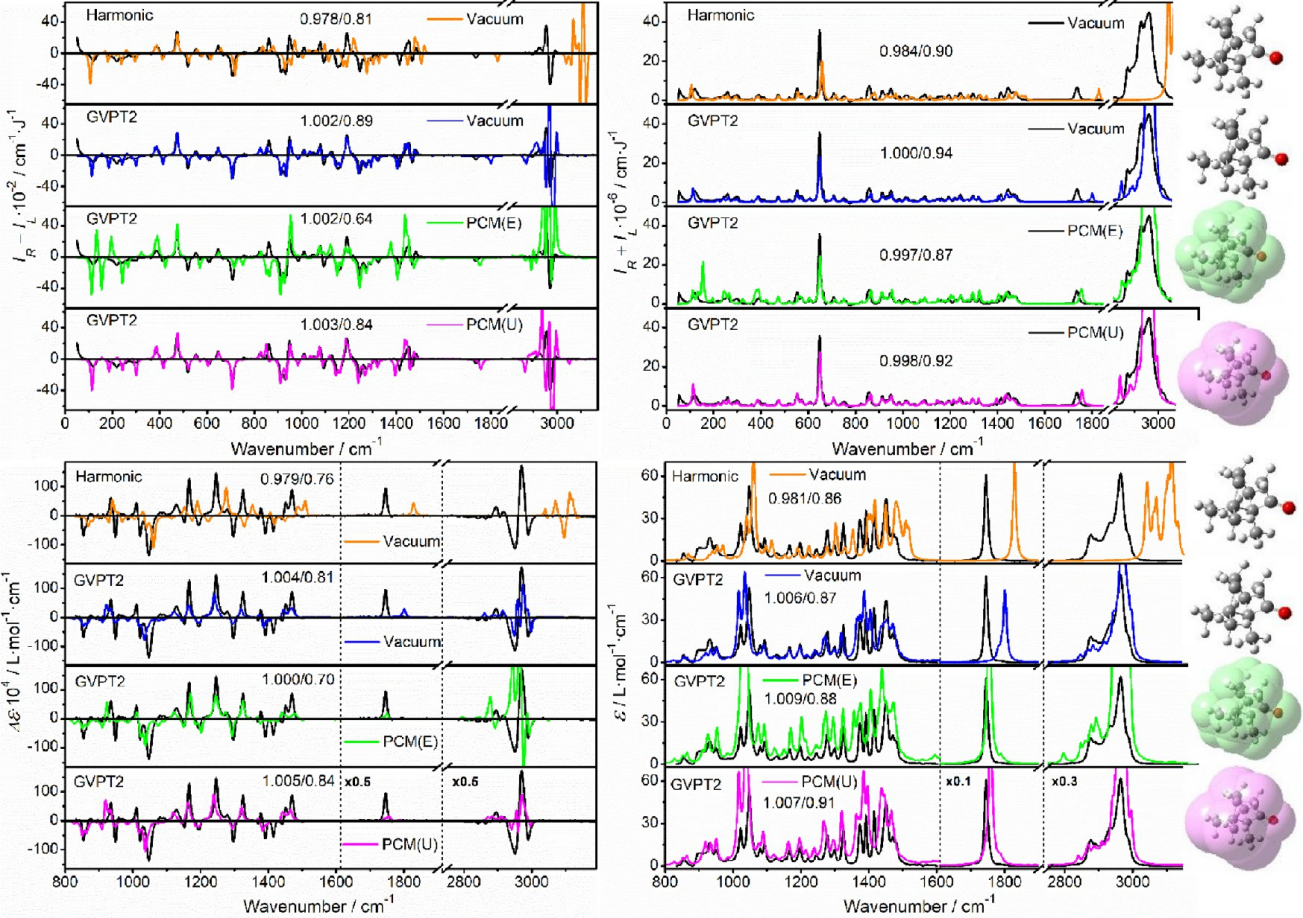
ROA, Raman, VCD, and IR spectra of (*R*)-camphor simulated at the B3PW91/jun-cc-pVTZ level, using the harmonic approximation in a vacuum (orange), and the GVPT2 theory in a vacuum (blue), with explicit hydrogen (E, green) and united atom (U, magenta) PCM dielectric cavities. Best frequency-scaling factors/similarities to the experiment (plotted in black, in CHCl_3_, idealized ″(*R*-*S*)/2″ ROA and VCD) are indicated.

### Higher Property Derivatives

To understand the problem, we analyzed the higher energy and derivatives defined above. Third and fourth energy derivatives calculated with various solvent schemes are remarkably similar (Table S2), which explains the similar GVPT2 performances for the frequencies seen in Figure 3. On the other hand, the default PCM cavities with explicit spheres around the hydrogen atoms appear to introduce unrealistically curved dependencies of the dipoles and polarizabilities on the reduced normal-mode coordinates. This is shown in Figure 4 for the selected third polarizability and dipole derivatives of (*R*)-camphor. While the first and second (Figure S2) derivatives calculated with and without PCM are reasonably close, the third derivatives deviate significantly. For many cases, the vacuum values at equilibrium (Δ*q* = 0) are small, while the default PCM makes them unreasonably large. This happens for the 1527 cm^–1^ mode, α-derivatives, the 997 cm^–1^ mode, α, *G*′, and A derivatives, or mode 1527 cm^–1^, μ derivatives. From the whole dependencies, we see that the curvatures of the property surfaces are very different for the default PCM compared to the vacuum and united atom calculations. Somewhat surprising may be the fact that the α-tensor is affected in the same way as *G*′ and A, because the latter two are supposed to be more sensitive to fine geometry changes.^4^ Similarly, the electric and magnetic dipoles are affected to the same extent, in spite of representing different orders of the electromagnetic perturbation to the molecular Hamiltonian.^39^

**Figure 4:**
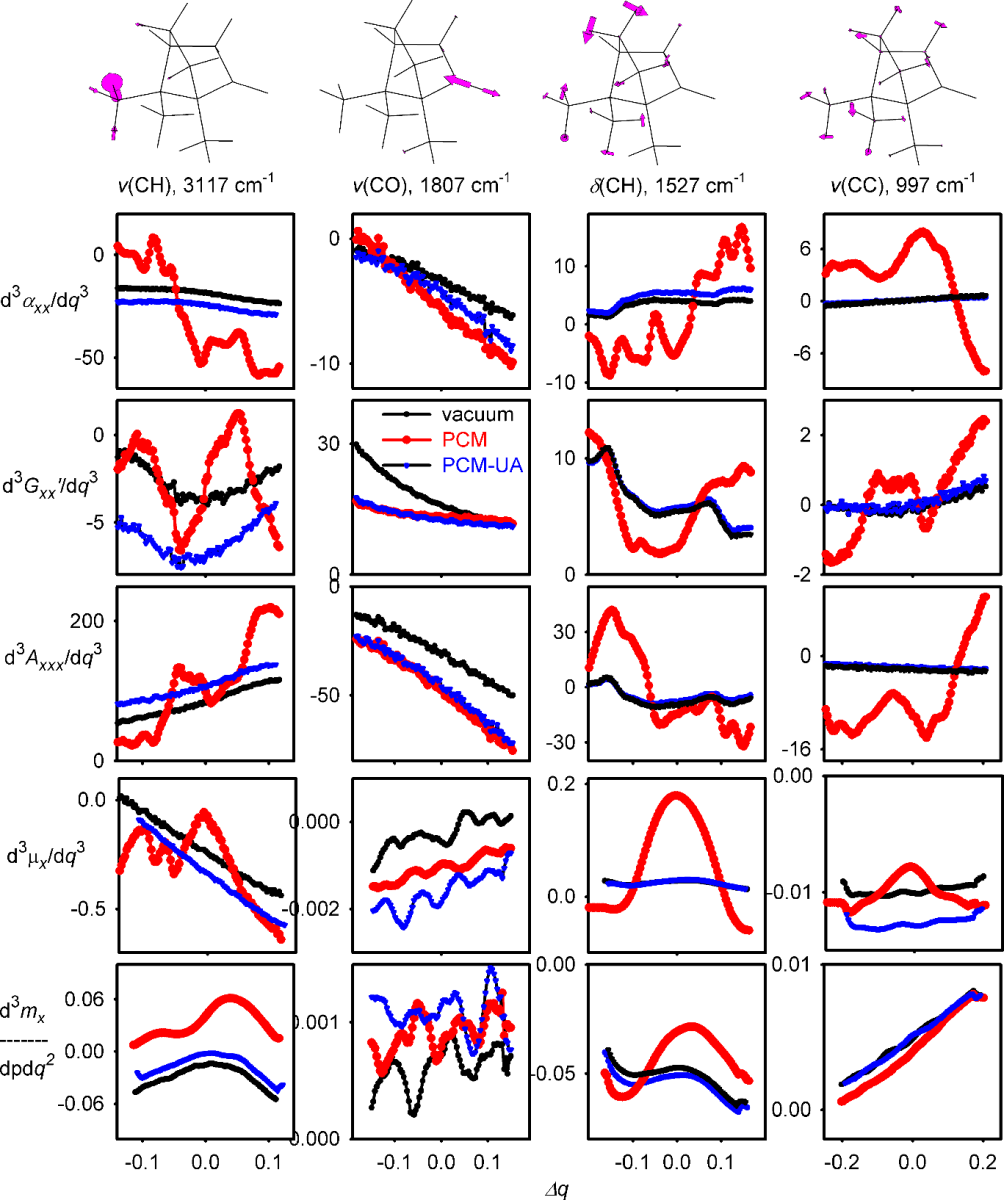
Camphor, third derivatives of α_*xx*_, *G*′_*xx*_, *A*_*x,xx*_, μ_*x*_, and *m*_*x*_ (in atomic units, B3LYP/6–311++G**) as dependent on the reduced normal-mode coordinates, for four vibrational normal modes shown at the top. Values obtained in vacuum (black) with the default (red) and united atom (blue) cavities are shown.

Another interesting aspect seen in Figure 4 is that the derivatives related to the C=O stretching motion (calculated at 1807 cm^–1^) are not affected much by PCM. This contrasts with motions including larger displacements of the hydrogen atoms, such as the 3117 cm^–1^ C–H stretching, or the 1527 cm^–1^ C–H bending. Similarly, the 997 cm^–1^ C–C stretching causes a deformation of the molecular cage and thus significant displacement of the hydrogens. These findings explain the “wrong sign” problem shown in Figure 3, since it predominantly occurs for vibrations with a significant participation of the hydrogen atoms. Only when the united atom cavities are used (blue curves in Figure 4), PCM and vacuum give closer results, meaning that the property surfaces are not unrealistically distorted by the cavity. This suggests that the united atom cavities should be preferably used for GVPT2 and similar models requiring higher-order property derivatives.

### Fine Computational Parameters

To investigate whether other conditions, except for the united cavities, may improve the PCM simulations, we simulated the (*R*)-camphor spectra with other variations. These included reduced dimensionality (Figure S3), solvent, functional, and basis set (Figure S4), and cavity modeling (Figure S5). The results are summarized in terms of spectral similarities in the low- and high-wavenumber regions and the number of wrong band signs in Table 1. Clearly, only the vacuum computation and PCM using the united atom cavity give reasonable results, confirming that the explicit hydrogen cavities remain the principal obstacle to the GPVT2 calculations.

**Table 1:** (*R*)-Camphor, VCD and ROA Spectra Calculated at Various Levels (Number of Wrong Band Signs (*N*) and Similarities (*s* in %) in Two Spectral Regions with Respect to Experiment)

	VCD				ROA			
	850–1550 cm^–1^		2800–3200 cm^–1^		80–1800 cm^–1^		2800–3200 cm^–1^	
model^a^	*N*	*s*	*N*	*s*	*N*	*s*	*N*	*s*
harmonic:								
(default)	10	19	4	–1	12	44	4	1
GVPT2:								
(default)	6	74	4	23	16	63	4	–39
SF	4	60	4	–38	11	45	3	–39
UF	4	60	4	–38	13	45	3	–39
SNSD	6	64	3	–32	12	47	4	–28
6–311++G(2d,p)	5	43	1	56	15	25	5	–21
B2PLYP, SNSD	5	66	4	–57	14	57	3	–30
CAM-B3LYP	7	47	4	–24	8	42	2	–37
HF	8	–4	4	–22	20	–7	5	2
**vacuum**	**1**	**74**	**0**	**66**	**0**	**88**	**1**	**29**
**UA0**	**1**	**72**	**0**	**65**	**0**	**79**	**1**	**56**
Pauling	7	61	1	74	25	13	1	–35
KS	8	40	4	–33	4	63	5	–58
B3PW91/CPCM	3	72	2	–40	2	75	2	2
*d* = 1	3	69	2	–10	0	80	2	43
*d* = 10	7	62	4	–48	26	21	3	14
*d* = 30	8	38	4	–41	28	–4	3	8

a The B3PW91/jun-cc-pVTZ/PCM level is used as a reference. Variations from it include: (1) the choice of the density integration grids, super fine (SF) and ultra fine (UF), (2) the basis sets, SNSD and 6–311++G­(2d,p) basis sets, (3) the B2PLYP, CAM-B3LYP, and HF functionals, (4) different representations of solvent effects by united atom cavities with atomic radii from universal force field (UA0) and Pauling atomic radii, radii from PBE0/6–31G­(d) calculation (KS), conductor-like solvent model (CPCM) and densities of integration points on the surface (*d*, in Å^2^, otherwise the default of 5 was used). The best combinations (vacuum and UA0) are in bold.

Still, variations of the parameters occasionally provide incremental improvements. An interesting phenomenon is the deterioration of the results when higher densities (*d* = 10, 30) of the integration points on the cavity surface are used compared to the default (*d* = 5). This is counterintuitive, as higher densities should lead to more accurate results, but it is in line with the better results for the united atom model with a smaller number of surface elements. Most probably, the cavity intersections, more “faithfully” described by the higher integration point densities, may also significantly contribute to the error of the electric properties.

Using the united atom cavities thus appears as a simple alternative when one wants to benefit from both the GVPT2 and PCM computational technologies. For the larger united cavities, fewer point charges and cavity elements change during the numerical differentiation, resulting in smaller changes in the derivatives. Another possibility for practical computations would be to calculate the GVPT2 wave functions and energies using the default PCM route and combine them with the third electric derivatives calculated in vacuum. Indeed, this option leads to similar results as when using the united cavities (Figure S6). It can also save the extra computational time needed to get the third electric derivative corrected for the environment. However, the savings are relatively small, and such a procedure complicates the computations.

### Real Effect of Third Electric Derivatives

In fact, the actual effect of the second and third electric derivatives (nondistorted by PCM) on the spectral patterns stemming from the fundamental modes is rather minor. This is documented in Figure 5, where camphor ROA spectra are simulated separately with contributions of first, first and second, and all polarizability derivatives included. As expected from the fundamental theory, where only the first derivatives contribute within the harmonic approximation, for the UA and vacuum models, almost no change in the spectra is observed when higher derivatives are included.

**Figure 5:**
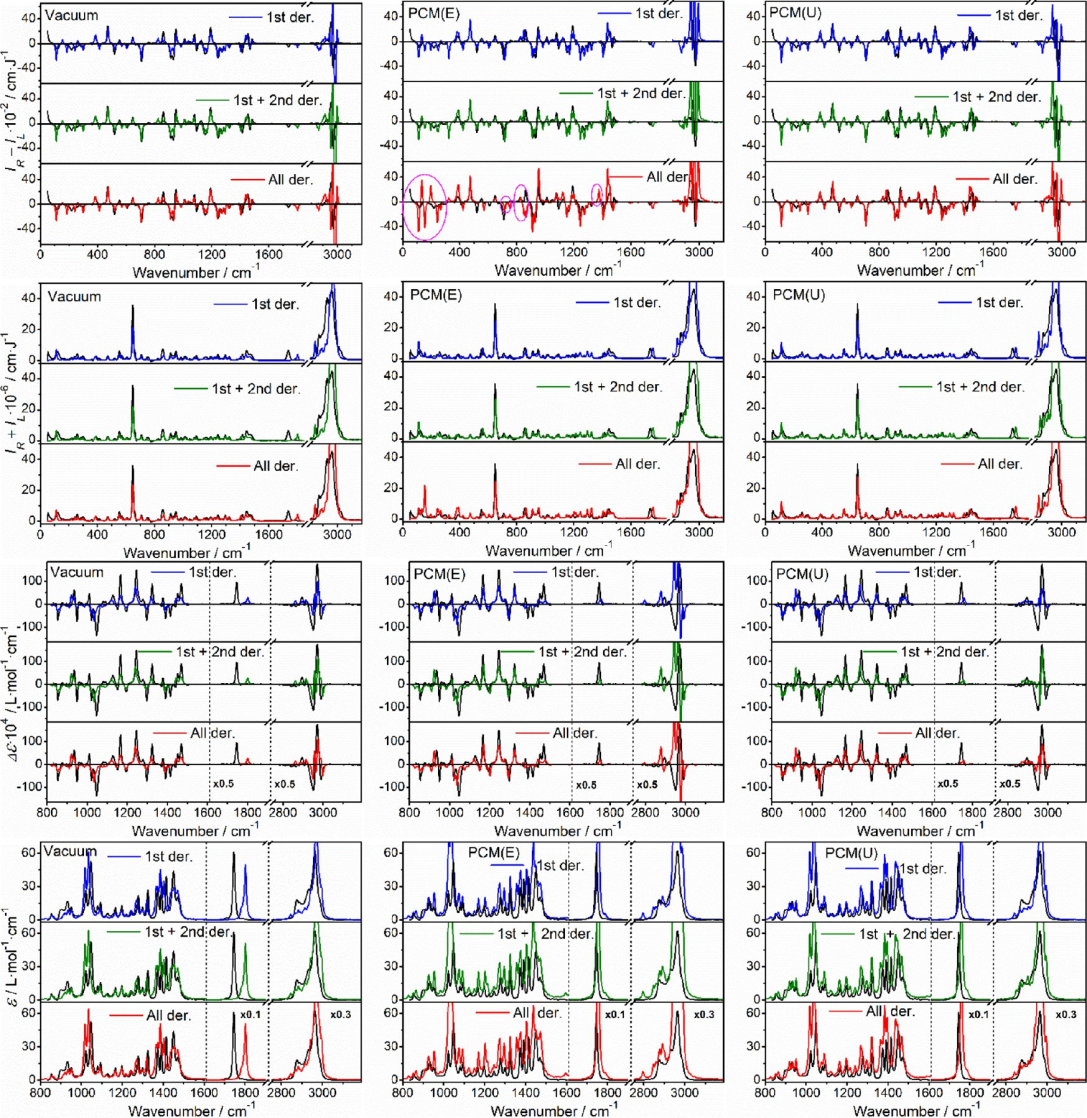
(*R*)-Camphor spectra calculated with first, first and second, and first, second, and third property derivatives. Top to bottom: ROA, Raman, VCD, and IR, left to right: vacuum, PCM with explicit (E) and unified (U) hydrogen cavities, GVPT2/B3PW91/jun-cc-pVTZ simulation. In the middle top panel, examples of wrongly predicted ROA signs are indicated by the magenta ellipses, and ideal (*R*-*S*)/2 ROA and VCD spectra are shown for the experiment (black lines).

However, the third derivative is still needed to reproduce in full the anharmonic spectral features. Their approximate average contribution can be estimated from the integrals plotted in Figure 6. Not surprisingly, intensities of the fundamental bands are governed by the first derivatives, except for the ROA/PCM­(E) case. However, the second and third derivatives appear also important for the fundamentals, on average accounting for ∼25% of the intensities. For ROA, the second and third derivatives contribute approximately equally in the vacuum and PCM­(U) cases.

**Figure 6:**
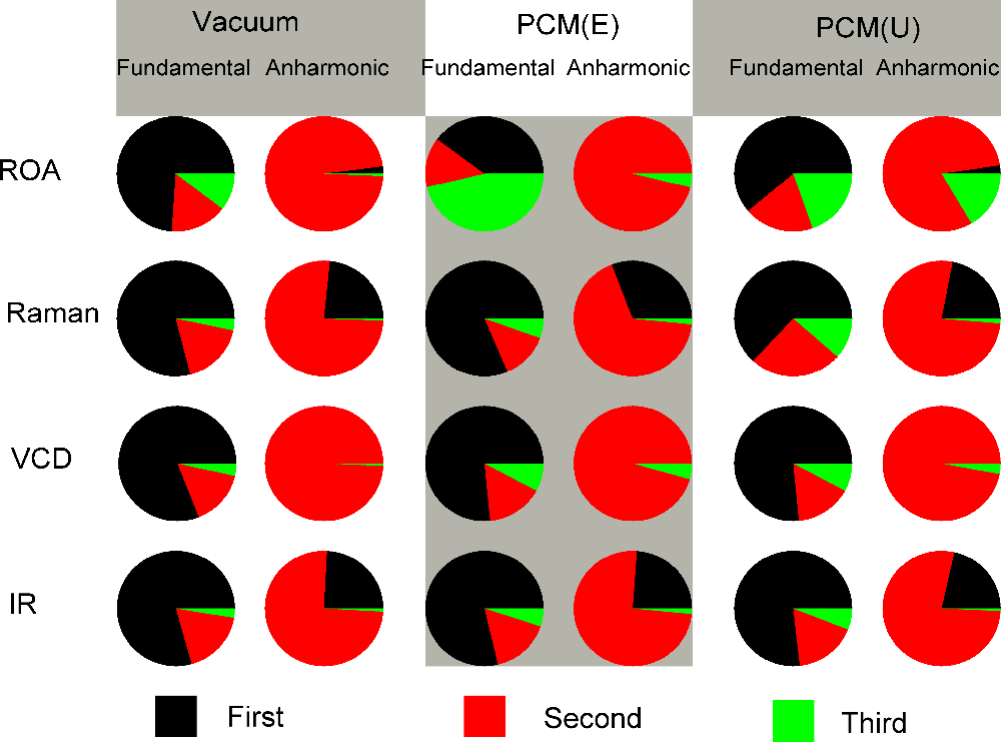
Approximate contributions of the first, second, and third property derivatives to spectral intensities, in the ″fundamental″ (0–2000 and 2750–3100 cm^–1^) and ″anharmonic″ (2000–2750 and 3100–4000 cm^–1^) regions. They were calculated as *n*(third) = (*I*_123_ −*I*_12_)/*I*_123_ × 100%, *n*(second) = (*I*_12_ – *I*_1_)/*I*_123_ × 100% and *n*(first) = 100 – *n*(third) – *n*(second), where the integrals are $I_{123}=\int |S_{123}|d\nu$ , where *S*_123_ is a spectrum generated using the first, second, and third derivatives, etc.

Regarding the region of the anharmonic bands, there is a striking difference between vibrational optical activity (ROA and VCD), the spectra of which arise almost exclusively from the second derivatives, and the parent Raman and IR spectra, where the first derivatives contribute as well, approximately by ∼25%. We also see small contributions from third property derivatives, not originally present in the formulas of the intensities for first overtones and combinations, which arise from the mixing with fundamentals through the variational correction. While the incidence is generally weak and consistent between vacuum and PCM models, we observe a higher variability in ROA, highlighting the sensitivity of this technique to minute adjustments in theory.

### Assignment of the Anharmonic Bands

The anharmonic parts of the four types of spectra of the three compounds are plotted in Figures 7–9. The assignment is based on the strongest transition contributing to selected bands and the leading term in the GVPT2 wave function, provided that it exceeds 80% of the total contributions. For each of the three molecules, the computation gives about 3000 vibrational transitions, from the ground state to monoexcited (fundamental), and double-excited combination and overtone states. At this point, we find it quite amazing that the present technologies allow one not only to measure such spectral details but also to simulate them up to almost band-to-band agreement. For example, on average, the calculated and experimental frequencies differ only by a few cm^–1^ (Tables S4–S6), and a majority of the relative band intensities, including the ROA and VCD signs, are reproduced.

**Figure 7:**
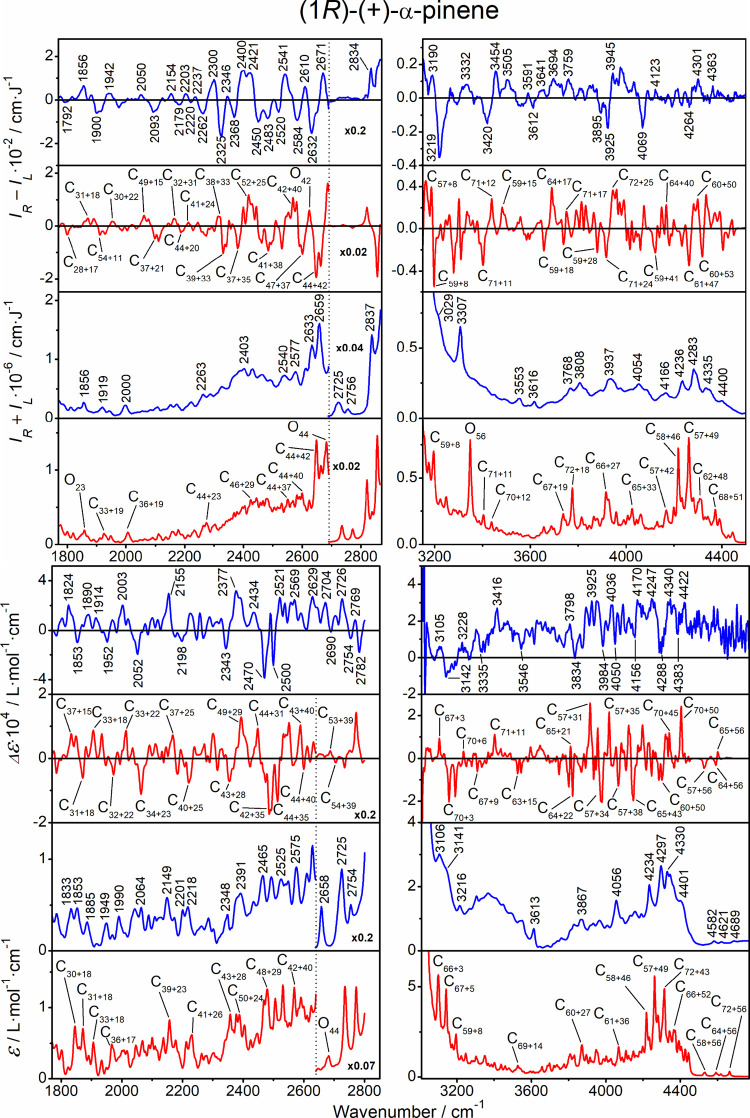
α-Pinene, ROA, Raman, VCD, and IR spectra in the anharmonic regions, experiment (blue, selected peak positions are indicated), and GVPT2 computation (red). For the bands in the latter, assignment of the most intense transitions is indicated [C–combination, O–overtone; the subscripts indicate participating modes listed in Table S3, ideal (*R*-*S*)/2 ROA and VCD experimental spectra are shown here and in Figures 8 and 9].

**Figure 8:**
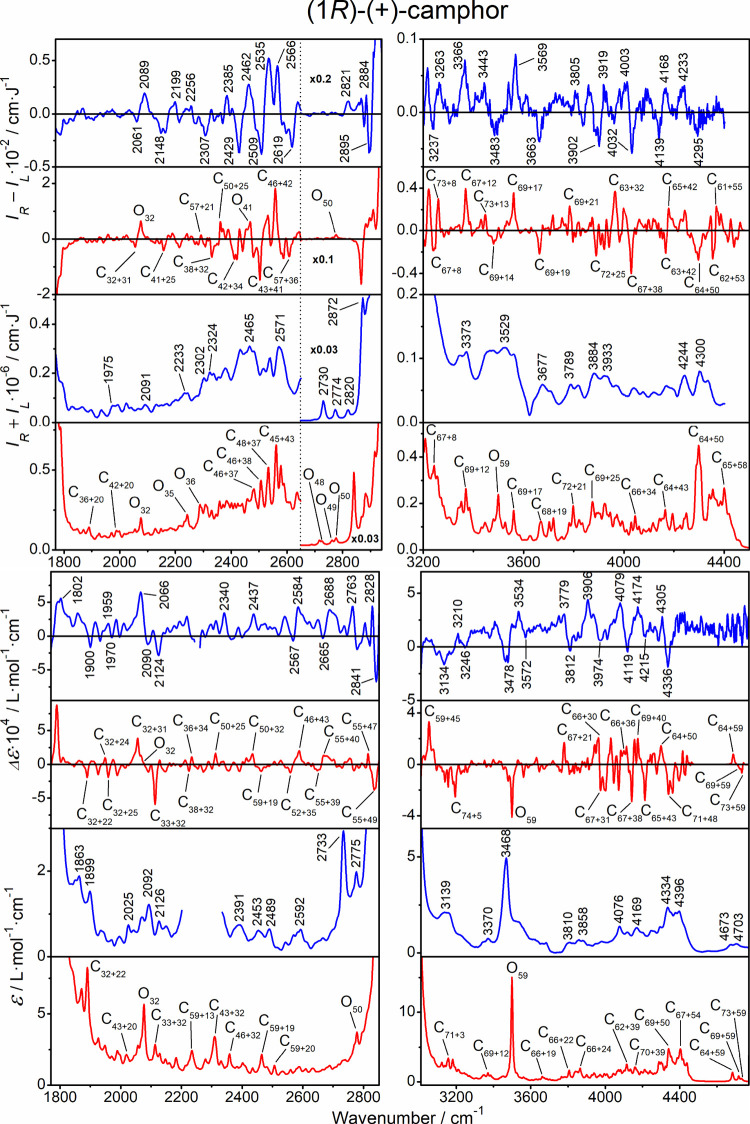
Camphor, ROA, Raman, VCD, and IR spectra in the anharmonic regions, experiment (blue, selected peak positions are indicated), and GVPT2 computation (red). For the bands in the latter, assignment of the most intense transitions is indicated as C–combination and O–overtone, and the subscripts indicate participating modes listed in Table S3.

**Figure 9:**
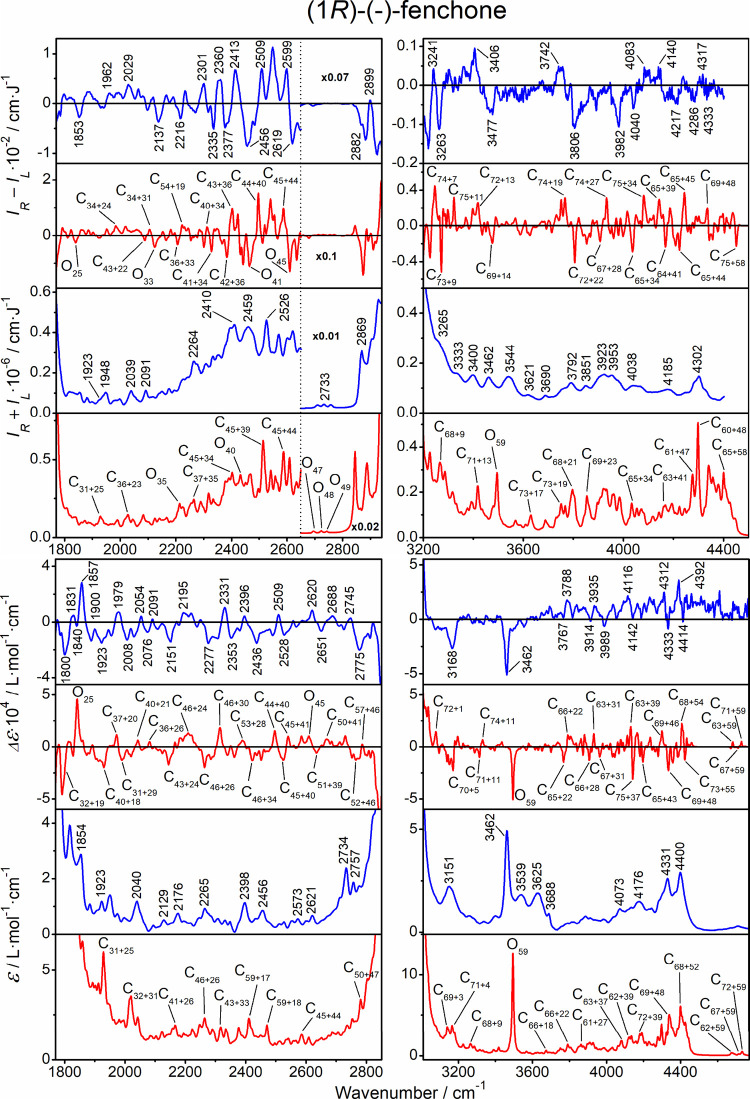
Fenchone, ROA, Raman, VCD, and IR spectra in the anharmonic regions, experiment (blue, selected peak positions are indicated), and GVPT2 computation (red). For the bands in the latter, assignment of the most intense transitions is indicated as C–combination and O–overtone; the subscripts indicate participating modes listed in Table S3.

The spectra carry a wealth of information about molecular mechanics and electromagnetic properties. From a more applied point of view, it may be interesting to note that the compounds form specific “fingerprints” in all kinds of vibrational spectra. For example, the transitions in the anharmonic region merge and are difficult to resolve in the Raman spectrum of α-pinene, while they are more structured for camphor and fenchone.

Vibrational optical activity and sign information immensely help to resolve and assign the spectral features. The overall quality of the simulations for ROA and VCD seems to be comparable. The computations also well-reproduce the *g*-factors (VCD/IR ratios) and circular intensity differences (ROA/Raman), both close to 10^–4^, only slightly lower than a typical value of the fundamental bands (∼2 × 10^–4^).

Most of the “anharmonic” bands can be assigned to double-excited combination transitions. With the current VPT2, we cannot calculate higher than twice excited states; however, since these describe most of the observed spectral features, we may suppose that transitions involving more than two quanta have a minimal impact on the spectrum. The anharmonic bands close to the fundamental C–H stretching bands pick up intensity from them. Occasional overtone bands have comparable intensities, but their number is obviously limited to the number of the fundamentals. Very distinct is the C–C stretching overtone in the Raman spectrum of α-pinene (O_56_, exp. at 3307 cm^–1^), and C–O stretching overtones in camphor and fenchone (O_59_, at 3468 and 3462 cm^–1^, respectively) in IR and VCD. Interesting ″silent regions″ appear in VCD and IR spectra within ∼4500–4700 cm^–1^ (ROA and Raman could not be measured this far). This matches the about 180–260 cm^–1^ separations between the C=C (α-pinene, 1660 cm^–1^) and C=O (camphor, fenchone, ∼1736 cm^–1^) stretching modes and the closest fundamental vibration (cage-deformation, ∼1480 cm^–1^).

## Conclusions

A unique set of high-quality reference data was obtained on which the generalized vibrational perturbation theory at the second order could be tested. Some of the anharmonic spectral features, in particular, in ROA, were observed for the first time. Vibrational optical activity ROA and VCD spectra greatly enhanced the assessment of the theoretical methods, as they are much more sensitive to fine computational details than the parent Raman/IR ones.

Rather surprisingly, the default PCM model with explicit cavities for all atoms appeared to be unsuitable for anharmonic simulations, even for the fundamental transitions. Further analysis showed that explicit cavities for hydrogen atoms introduce unrealistic perturbations into the dependence of electric and magnetic dipoles and the Raman/ROA polarizabilities on the geometry. In particular, the third property derivatives appeared to be quite unusable for the GVPT2 method. Fortunately, the problem could be rectified using united atom cavities or by a combination of computations performed at different levels. Other computational parameters had a limited effect on the accuracy.

The improved computational protocol then provided an excellent computational basis for the interpretation of the vibrational spectra, reproducing frequencies, relative intensities, and signs of the observed bands with high fidelity. The “black-box” GVPT2 computational methodology, coupled with sensitive spectrometers, proved to be able to produce amazing details about molecular properties, potentially useful in analytical chemistry and material science.

## Supplementary Material



## Notes

Experimental spectra of camphor in CHCl_3_ and MeOH, examples of polarizability second derivatives, further computational tests, experimental conditions, and assignments of selected vibrational bands (PDF)
