# Supplementary material for: Black-Box Simulations of Anharmonic Vibrational Chiroptical Spectra: Problems with Property Third Derivatives and the Solvent
Source: J Chem Theory Comput. 2025 Oct 13;21(20):10489–502. doi: 10.1021/acs.jctc.5c01132 (PMC12573746; doi:10.1021/acs.jctc.5c01132)
Supplement: Supplementary file 1 [file ct5c01132_si_001.pdf]

Supporting Information

Black-Box Simulations of Anharmonic Vibrational Chiroptical Spectra: Problems with Property Third Derivatives and the Solvent

Qin Yang,[†] Valery Andrushchenko,[†] Jana Hudecová,[‡] Josef Kapitán,[‡] Julien Bloino,[#] Isabelle Bowker,[†] and Petr Bouř[†]

[†] Institute of Organic Chemistry and Biochemistry, Academy of Sciences, Flemingovo náměstí 2, 16610, Prague, Czech Republic

[‡] Department of Optics, Palacký University Olomouc, 17. listopadu 12, 77900, Olomouc, Czech Republic

[#] Scuola Normale Superiore di Pisa, Piazza dei Cavalieri 7, 56126 Pisa, Italy

Contents

(R)-camphor test:

Figure S1. Experimental raw ROA and Raman spectra of camphor in $CHCl_3$ and MeOH.

Figure S2. Examples of polarizability second derivatives.

Figure S3. Spectra calculated with various number of low-frequency modes fixed.

Figure S4. Spectra calculated at various approximations.

Figure S5. Spectra calculated for different solvent models.

Figure S6. Spectra calculated combining different levels.

Tables:

Table S1. Details of Raman and ROA experiments.

Table S2. Comparison of third and fourth energy derivatives calculated in vacuum and PCM.

Table S3. Mode assignment.

Table S4. α-Pinene, selected transitions, including anharmonic bands.

Table S5. Camphor, selected transitions, including anharmonic bands.

Table S6. Fenchone, selected transitions, including anharmonic bands.

Gaussian input examples

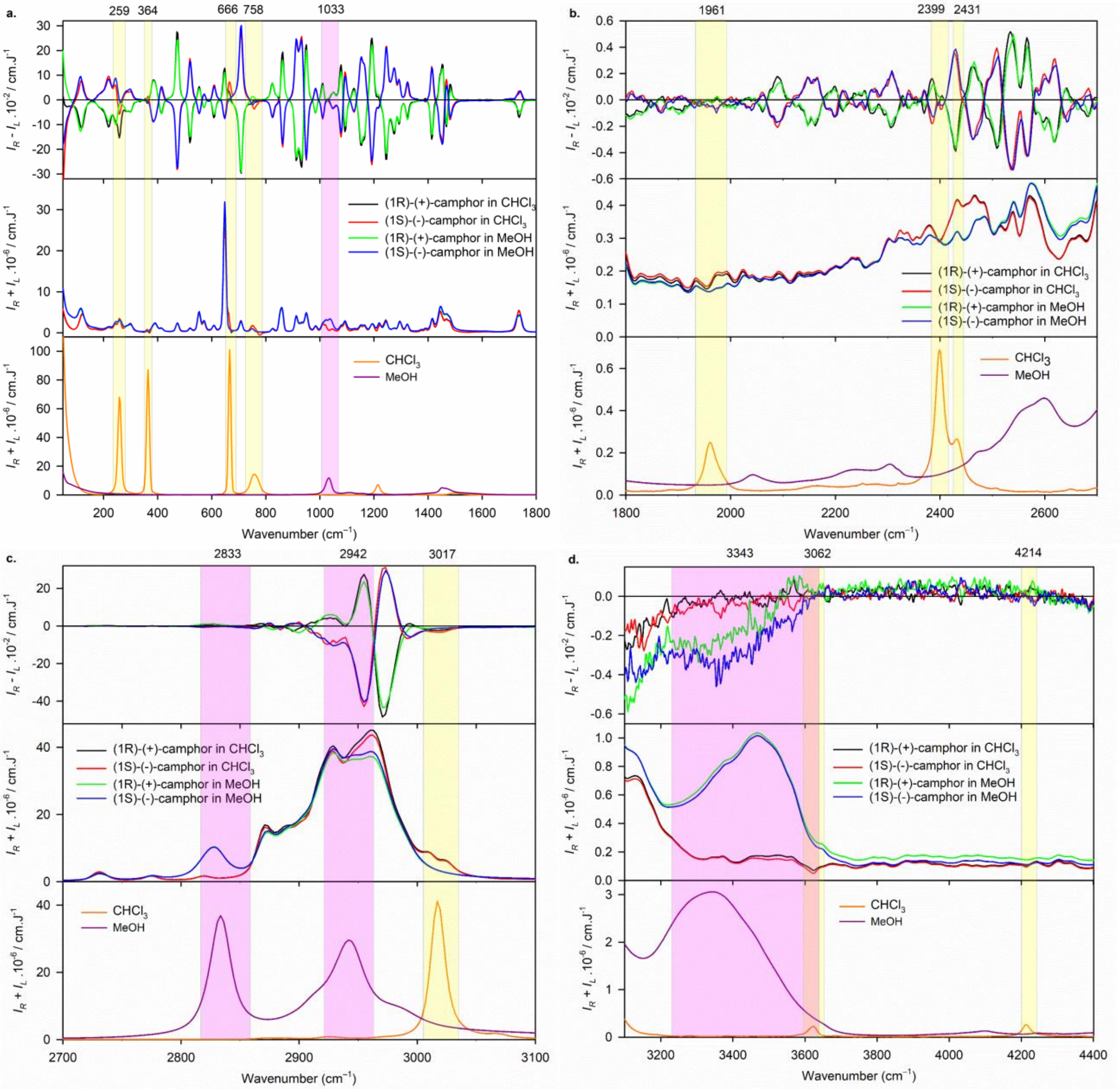

Figure S1. Experimental raw ROA (top) and Raman (middle) spectra of camphor in $CHCl_3$ and MeOH in (a) fingerprint 50-1800 cm^{-1}, (b) low combination and overtone 1800-2700 cm^{-1}, (c) C-H stretching 2700-3100 cm^{-1}, and (d) high combination and overtone 3100-4400 cm^{-1} regions. Raman spectra of solvents are plotted at the bottom; their signal was subtracted from Raman spectra, strongest solvent bands cause minor artifacts in ROA spectra (marked). No background correction was applied for this Figure.

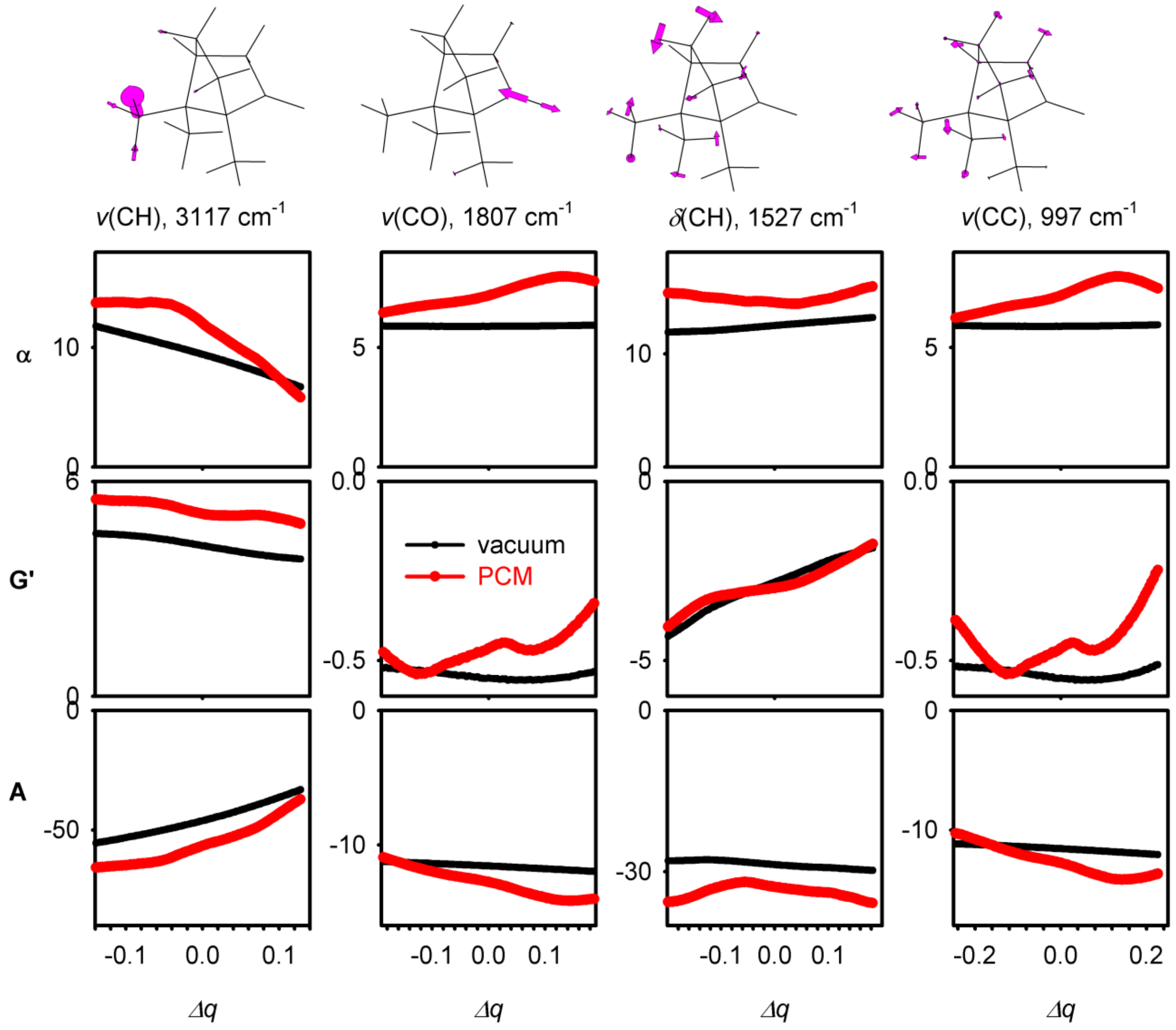

Figure S2. Camphor, normal mode second derivatives (α_{xx},G'_{xx}, $A_{x,xx}$, in atomic units) as dependent on the mode shift (dimensionless reduced coordinates), B3LYP/6-311++G**, default explicit hydrogen PCM cavity.

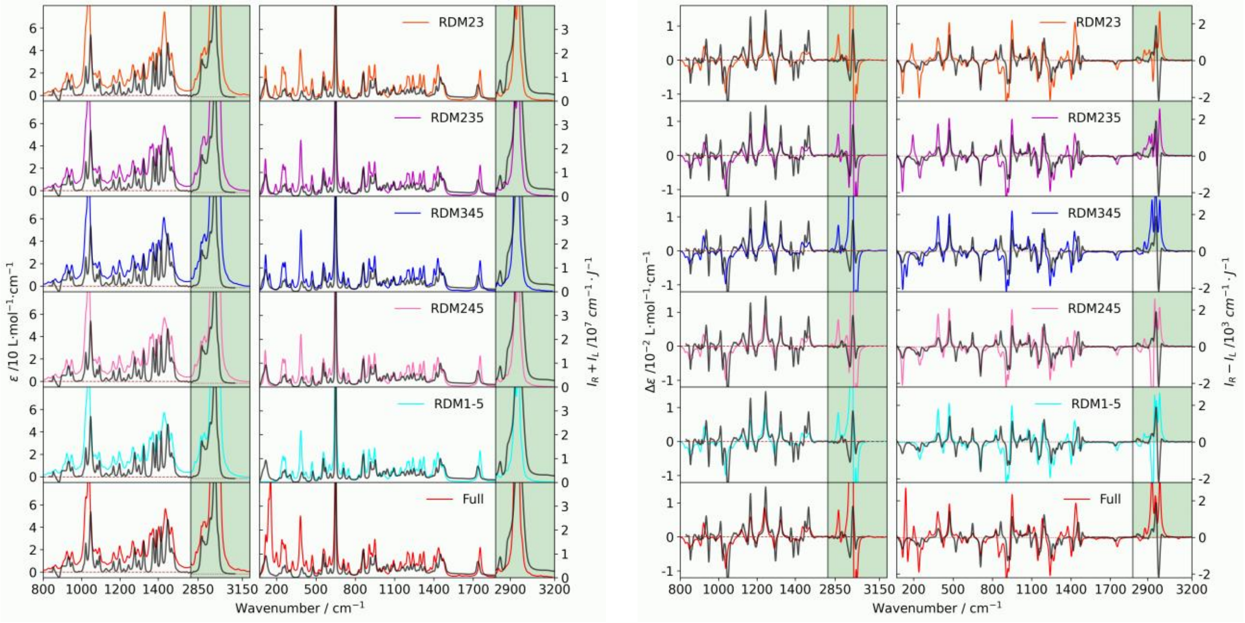

Figure S3. (R)-camphor, IR and VCD ($\varepsilon/\Delta\varepsilon$ in $10/10^{-2}$ $Lmol^{-1}cm^{-1}$), Raman and ROA ($I_R + I_L$, $I_R - I_L$, computed intensities multiplied by a constant to fit the experiment). Spectra simulated using five schemes dealing with large amplitude motions: 2 and 3 hindered rotor modes frozen (RDM23), modes 2,3 and 5 frozen (RDM235), etc., all dimensional calculation (full), B3PW91/jun-cc-pVTZ/PCM calculation.

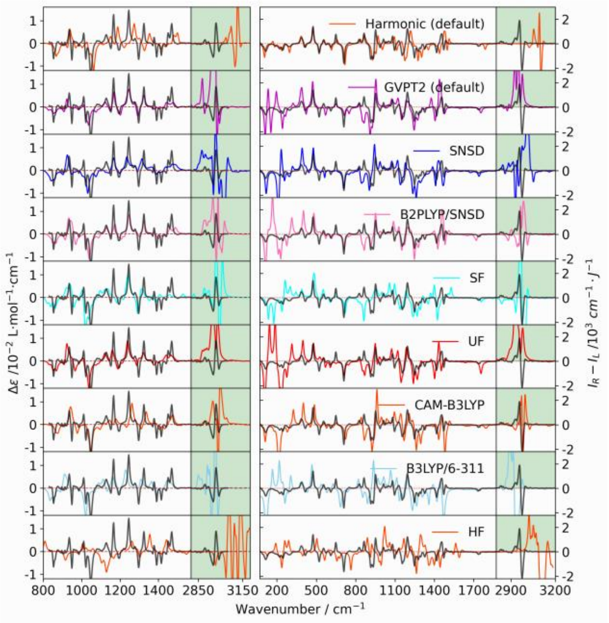

Figure S4. (*R*)-camphor, VCD and ROA. Simulations with various combinations of the harmonic and anharmonic levels. The B3PW91/jun-cc-pVTZ/PCM level was used as default, indicated variations include super fine (SF) and ultra fine (UF) grids, the SNSD and 6-311++G(2d,p) (6-311) basis sets, B2PLYP, B3LYP, CAM-B3LYP and HF functionals.

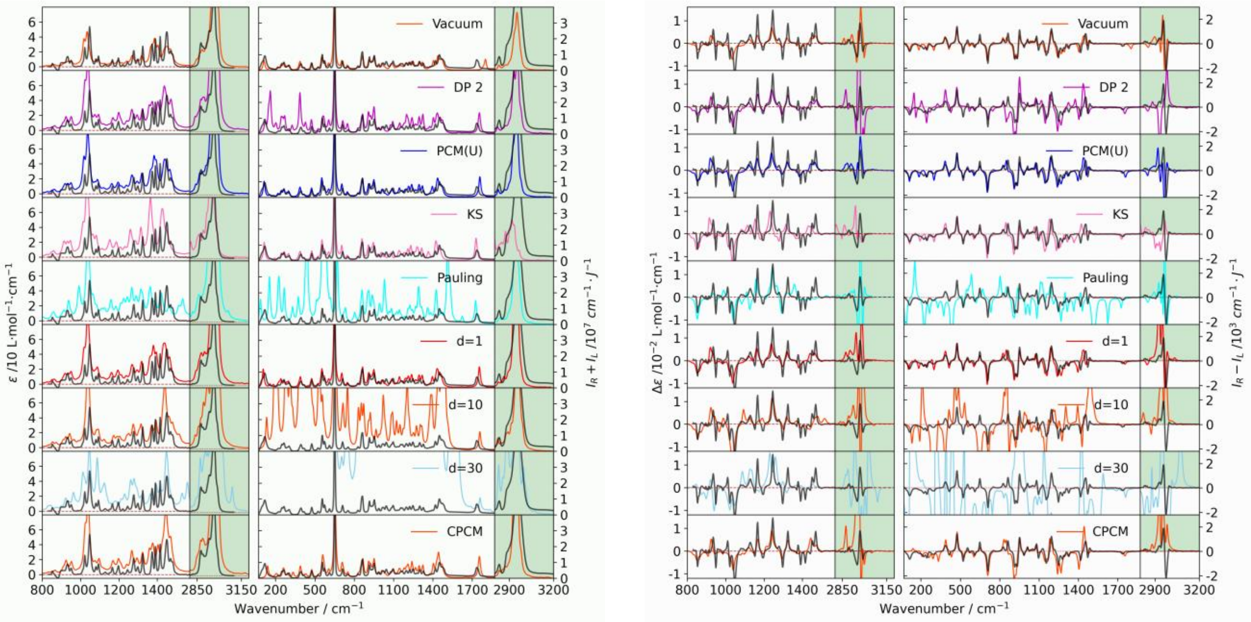

Figure S5. (R)-camphor, IR, Raman, VCD, and ROA spectra simulated at the anharmonic B3PW91/jun-cc-pVTZ level, in vacuum, PCM with the dynamic permittivity (DP) of 2, united atom cavity with atomic radii from universal force field [PCM(U)] and PBE0/6-31G(d) calculation (KS), Pauling atomic radii, 1, 20 and 30 integration point densities (default is $d = 5$ Å^{-2}), and with the conductor-like solvent model (CPCM).

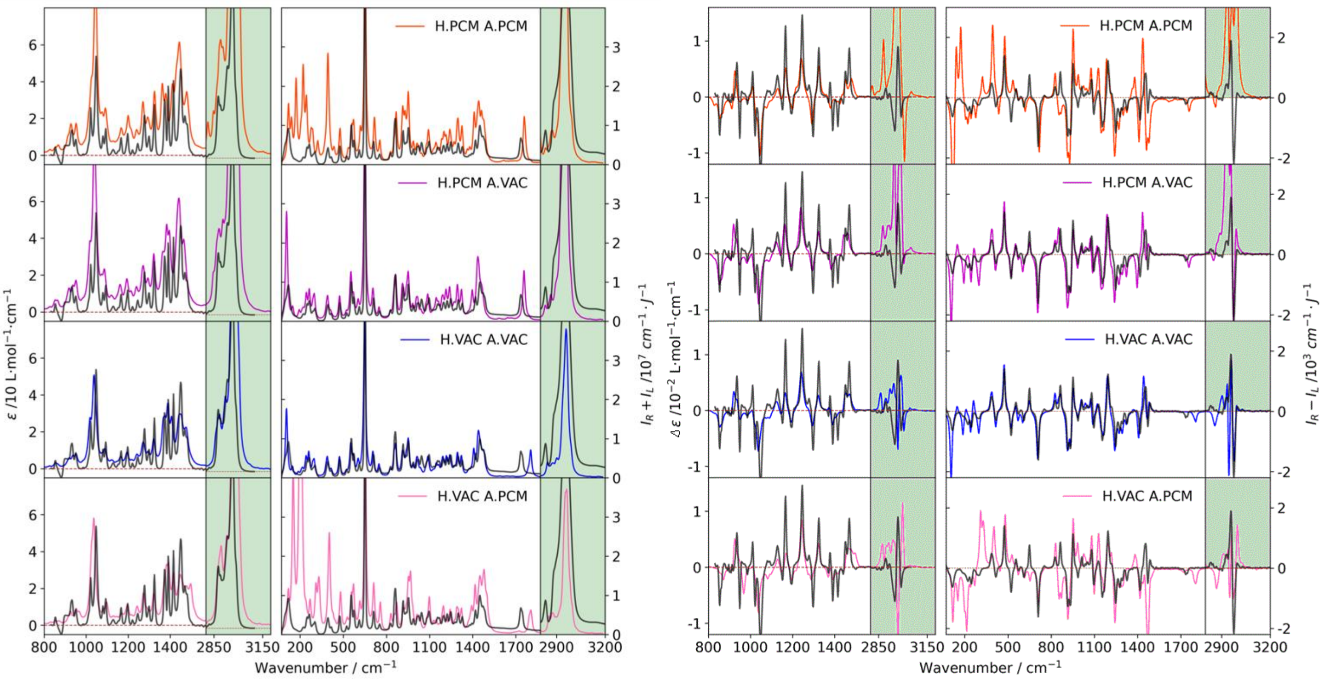

Figure S6. IR. Raman, VCD, and ROA camphor spectra calculated by combining harmonic (H) and anharmonic (A) constants in gas and PCM:

	geometry and normal modes	anharmonic constants
H.PCM A.PCM	PCM	PCM
H.PCM A.GAS	PCM	vacuum
H.GAS A.GAS	vacuum	vacuum
H.GAS A.PCM	vacuum	PCM

Table S1. Raman and ROA experiments: laser power at the sample (*P*) and total accumulation time (*t*). Values with asterisks (*P**, *t**) apply for measurements of weak combination and overtone bands

compound	enantiomer	*P* / mW	t / h	P* / mW	t* / h
camphor in $CHCl_3$	R	74	3.6	240	41.4
	S	74	3.5	240	40.8
camphor in MeOH	R	240	2	240	32.8
	S	240	2.2	240	41.3
α-pinene	R	406	1.1	406	12.5
	S	210	0.4	406	26.4
fenchone	R	240	2.2	240	36.4
	S	240	2	240	40.8

Table S2. (*R*)-camphor, comparison of all third and fourth energy derivatives in Cartesian coordinates, calculated at the B3LYP/6-311++G** level in vacuum, using the united atom [PCM(U)] and default [PCM(E)] model, with water and methanol as solvents. Average deviations multiplied by 10^4 are given, in atomic units. (Note that the correlation coefficients would be close to one, to about 5 decimal figures.)

Third Energy Derivatives × 10^4

	vacuum	UA/H_2O	PCM/H_2O	PCM/MeOH
vacuum	0.0	4.3	5.1	5.0
UA/H_2O		0.0	1.5	1.5
PCM/H_2O			0.0	0.1
PCM/MeOH				0.0

Fourth Energy Derivatives × 10^4

	vacuum	UA/H_2O	PCM/H_2O	PCM/MeOH
vacuum	0.0	7.3	7.4	7.2
UA/H_2O		0.0	7.6	7.6
PCM/H_2O			0.0	1.1
PCM/MeOH				0.0

Table S3. Mode numbering for Tables S4-S6 and Figures 7-9, harmonic frequencies ν in cm^{-1}, B3LYP/jun-cc-pVTZ/PCM(UA)/G3BJ computations.[a]

	Fenchone		Camphor		α-Pinene	
	ν		ν		ν	
1	80	τ(CC)	109	τ(CC)	120	τ(CC)
2	178	τ(CC)	170	τ(CC)	196	τ(CC)
3	187	τ(CC)	185	τ(CC)	206	τ(CC)
4	198	τ(CC)	217	τ(CC)	217	τ(CC)
5	206	τ(CC)	224	τ(CC)	239	τ(CC)
6	224	τ(CC)	238	τ(CC)	268	τ(CC)
7	256	τ(CC)	260	τ(CC)	309	τ(CC)
8	288	τ(CC)	290	τ(CC)	339	τ(CC)
9	296	τ(CC)	297	τ(CC)	392	τ(CC)
10	321	τ(CC)	381	τ(CC)	400	τ(CC)
11	366	τ(CC)	389	τ(CC)	433	τ(CC)
12	420	τ(CC)	410	τ(CC)	470	τ(CC)
13	442	τ(CC)	474	τ(CC)	492	τ(CC)
14	509	τ(CC)	523	τ(CC)	582	τ(CC)
15	523	τ(CC)	559	τ(CC)	632	τ(CC)
16	601	τ(CC)	577	τ(CC)	683	τ(CC)
17	667	τ(CC)	615	τ(CC)	792	τ(CC)
18	727	τ(CC)	658	τ(CC)	815	τ(CC)
19	789	τ(CC)	718	τ(CC)	837	τ(CC)
20	829	τ(CC)	761	τ(CC)	862	τ(CC)
21	844	τ(CC)	836	τ(CC)	908	τ(CC)
22	848	τ(CC)	867	τ(CC)	930	τ(CC)
23	902	τ(CC)	880	τ(CC)	950	τ(CC)
24	907	τ(CC)	928	τ(CC)	963	τ(CC)
25	946	τ(CC)	938	τ(CC)	979	τ(CC)
26	957	τ(CC)	955	τ(CC)	985	τ(CC)
27	963	τ(CC)	958	τ(CC)	1028	τ(CC)
28	972	τ(CC)	969	τ(CC)	1043	τ(CC)
29	1003	τ(CC)	1004	τ(CC)	1068	τ(CC)
30	1013	τ(CC)	1029	τ(CC)	1070	τ(CC)
31	1033	τ(CC)	1040	τ(CC)	1091	τ(CC)
32	1037	τ(CC)	1063	τ(CC)	1117	τ(CC)
33	1086	τ(CC)	1095	τ(CC)	1134	τ(CC)
34	1119	τ(CC)	1113	τ(CC)	1159	τ(CC)
35	1136	τ(CC)	1151	τ(CC)	1200	τ(CC)
36	1175	τ(CC)	1175	τ(CC)	1219	τ(CC)
37	1191	τ(CC)	1189	τ(CC)	1241	τ(CC)
38	1220	τ(CC)	1216	τ(CC)	1251	τ(CC)
39	1237	τ(CC)	1224	τ(CC)	1260	τ(CC)
40	1250	τ(CC)	1246	τ(CC)	1288	τ(CC)
41	1263	τ(CC)	1268	τ(CC)	1306	τ(CC)
42	1271	τ(CC)	1275	τ(CC)	1347	τ(CC)
43	1294	τ(CC)	1303	τ(CC)	1369	τ(CC)
44	1316	τ(CC)	1325	τ(CC)	1378	τ(CC)
45	1343	τ(CC)	1330	τ(CC)	1407	δ(HCH)
46	1363	τ(CC)	1354	τ(CC)	1416	δ(HCH) δ(HCC)
47	1384	δ(HCH)	1396	δ(HCH)	1425	δ(HCH)
48	1404	δ(HCH)	1403	δ(HCH)	1477	τ(CC) δ(HCH)
49	1407	δ(HCH)	1416	δ(HCH)	1480	δ(HCH)
50	1470	τ(CC)	1441	δ(HCH) τ(C-C)	1485	τ(CC)
51	1474	τ(CC)	1471	τ(CC)	1488	τ(CC) δ(HCH)
52	1478	τ(CC)	1476	τ(CC)	1495	τ(CC)
53	1481	τ(CC)	1481	τ(CC)	1502	τ(CC)

54	1486	τ(CC)	1485	τ(CC)	1517	δ(HCH)
55	1488	τ(CC)	1491	τ(CC)	1521	τ(CC)
56	1494	τ(CC)	1501	τ(CC)	1723	ν(C=C)
57	1500	δ(HCH)	1504	τ(CC)	2985	ν(CH)
58	1518	τ(CC)	1513	τ(CC)	2998	ν(CH)
59	1790	ν(C=O)	1791	ν(C=O)	3002	ν(CH)
60	3038	ν(CH)	3037	ν(CH)	3010	ν(CH)
61	3039	ν(CH)	3040	ν(CH)	3017	ν(CH)
62	3043	ν(CH)	3042	ν(CH)	3035	ν(CH) τ(CC)
63	3053	ν(CH)	3059	ν(CH)	3035	ν(CH)
64	3062	ν(CH)	3061	ν(CH)	3046	ν(CH)
65	3069	ν(CH)	3069	ν(CH)	3057	ν(CH)
66	3091	ν(CH)	3093	ν(CH)	3058	ν(CH)
67	3095	ν(CH)	3096	ν(CH)	3064	ν(CH)
68	3109	ν(CH)	3099	ν(CH)	3086	ν(CH)
69	3111	ν(CH)	3103	ν(CH)	3093	ν(CH)
70	3111	ν(CH)	3104	ν(CH)	3107	ν(CH)
71	3112	ν(CH)	3111	ν(CH)	3113	ν(CH)
72	3115	ν(CH)	3113	ν(CH)	3129	ν(CH)
73	3119	ν(CH)	3114	ν(CH)	-	-
74	3126	ν(CH)	3116	ν(CH)	-	-
75	3127	ν(CH)	3134	ν(CH)	-	-

[a] automatic assignment based on relative coordinate changes, ν - stretching, δ - bending, τ - torsion, for orientation only, please see other works for a more detailed assignment (e.g., Longhi, G.; Abbate, S.; Gangemi, R.; Giorgio, E.; Rosini, C. Fenchone, Camphor, 2-Methylenefenchone and 2-Methylenecamphor: A Vibrational Circular Dichroism Study. *J. Phys. Chem. A* **2006**, *110*, 4958−4968, https://doi.org/10.1021/jp057178y, and Bouř, P.; Baumruk, V.; Hanzlíková, J. Measurement and Calculation of the Raman Optical Activity of Alpha-pinene and Trans-pinane *Collect. Czech. Chem. Commun.* **1997**, *62*, 1384−1395, https://doi.org/10.1135/cccc19971384).”).

.

Table S4.: α-Pinene. Calculated transition frequencies (ν, cm^{-1}), dipole and rotational strengths, Raman and ROA intensities (absolute values of *D*, *R*, *Ram*, and *ROA*, in % of the maximal value), type of transition (F-fundamental, O-overtone, C-combination), and involved modes. Strongest 200 transitions are listed, Experimental frequencies (ν_{exp}, cm^{-1}) are provided for selected transitions.

ν	ν_{exp}	*D*	*R*	*Ram*	*ROA*			
106		58.4	-5.8	1.3	0.3	F	1	
182		1.0	6.5	0.2	-0.1	F	3	
194		2.0	-3.3	0.3	-0.5	F	2	
207		3.9	0.3	0.1	0.2	F	4	
224		3.3	13.9	0.2	-1.1	F	5	
265		1.9	4.3	1.1	-0.6	F	6	
310		14.8	-19.9	0.9	0.4	F	7	
339		21.3	-15.8	0.2	-0.1	F	8	
389		0.7	1.2	0.8	2.7	F	9	
400		4.7	0.3	0.4	1.3	C	5	3
400		2.0	0.2	0.4	0.5	F	10	
432		13.1	0.3	0.5	0.6	F	11	
469		2.7	-0.4	1.0	0.5	F	12	
487		4.2	-8.1	1.0	-1.8	F	13	
571		3.7	2.1	0.7	0.9	C	12	1
575		12.0	7.2	1.9	2.6	F	14	
625		8.7	-17.8	1.2	-2.6	F	15	
671		0.5	2.7	8.3	-2.8	F	16	
680		0.2	0.7	1.0	-0.4	O	8	
775		12.6	14.8	1.7	-5.1	F	17	
778		4.9	5.3	0.7	-1.8	C	12	7
780		7.0	7.7	0.6	-2.0	O	9	
789		0.9	0.3	0.1	-0.1	C	10	9
800		93.5	1.2	0.4	9.6	F	18	
806		2.3	0.3	0.0	0.1	C	12	8
815		0.5	1.9	0.3	0.8	C	15	2
819		2.9	10.7	2.5	4.5	F	19	
838		0.2	0.5	0.3	0.9	C	14	6
844		3.7	7.2	4.5	1.7	F	20	
888		7.2	-1.3	0.5	3.1	C	15	6
890		15.4	2.6	1.0	5.3	F	21	
906		1.4	1.7	0.2	-0.1	C	18	1
910		3.6	12.5	2.7	-2.9	F	22	
931		4.6	7.9	1.2	-3.4	F	23	
946		2.6	-1.9	3.0	1.7	F	24	
959		24.0	-9.8	3.0	-18.4	F	25	
964		2.3	-0.8	0.3	-0.7	C	14	9
966		2.2	4.2	1.2	5.3	F	26	
973		0.9	-0.8	0.1	-0.2	O	13	
1004		2.2	-37.6	1.0	-0.3	F	27	
1008		1.3	0.8	0.0	0.0	C	18	4
1023		24.3	23.8	2.2	-3.8	F	28	
1034		1.1	0.0	0.1	-0.2	C	20	2
1041		0.2	0.7	0.0	0.0	C	14	12
1041		1.1	-0.2	0.4	-0.5	C	19	5
1044		1.9	-0.5	1.1	0.4	F	29	
1046		5.0	6.3	3.1	-0.5	F	30	
1051		0.7	2.1	0.3	0.8	C	20	4
1071		9.5	-15.0	0.9	4.0	F	31	
1092		15.1	9.1	4.3	-2.1	F	32	
1102		1.2	-4.1	0.0	0.2	C	22	2
1105		7.4	-27.7	0.3	2.3	F	33	
1112		0.8	-1.2	0.1	-0.3	C	21	5
1133		18.7	36.8	4.4	5.1	F	34	
1139		0.9	1.5	0.2	0.1	C	18	8
1149		0.2	0.9	0.0	0.1	O	14	
1158		0.4	0.8	0.1	0.0	C	19	8
1172		2.4	0.3	1.2	-1.8	C	26	4
1174		6.6	2.2	3.8	-6.0	F	35	
1183		2.3	-5.2	1.6	1.0	C	26	5
1190		1.2	0.2	0.2	-0.5	C	27	3
1192		5.5	-4.7	2.9	2.8	F	36	
1199		0.8	0.4	0.1	-0.4	C	15	14
1209		13.7	-3.2	2.2	-1.8	F	37	
1217		5.3	-20.1	0.9	4.5	F	38	
1220		1.6	-0.2	0.4	-0.6	C	22	7
1226		3.2	-6.1	0.6	0.1	C	27	5
1229		11.4	1.5	2.4	-2.6	F	39	
1231		2.8	4.3	0.9	-1.0	C	26	6
1244		0.1	0.8	0.1	0.0	C	17	12
1251		1.1	-0.6	0.0	-0.2	C	29	4
1267		12.3	-13.8	6.8	-4.2	F	41	
1274		2.0	-0.5	1.2	-1.0	C	20	11
1299		0.4	1.6	0.0	0.1	C	25	8
1308		0.8	0.7	0.9	0.3	C	29	6
1315		2.8	4.9	2.9	-0.1	F	42	
1334		11.5	18.3	7.6	-5.8	F	43	
1344		2.2	2.3	0.8	-0.9	O	16	
1344		5.0	-6.3	1.6	3.3	F	44	
1374		27.7	-4.0	0.2	-0.1	F	45	
1378		6.9	-1.4	0.1	-0.3	C	22	12
1383		10.2	-0.4	8.6	-1.8	F	46	
1394		21.0	-6.7	0.3	-0.2	F	47	
1412		0.9	-0.2	0.1	0.0	C	24	12
1431		2.6	1.1	0.1	-0.8	C	26	12
1432		9.5	-0.4	1.3	-1.1	C	29	9
1435		2.4	-0.2	2.0	1.4	C	30	9
1435		10.6	2.3	6.4	7.0	C	35	6
1438		7.9	0.1	6.3	4.8	C	35	6
1439		14.0	0.0	2.1	4.8	F	48	
1443		14.4	5.1	1.0	-4.9	F	50	
1444		0.7	-0.2	0.2	0.5	C	29	10
1444		0.7	0.9	0.4	0.6	C	33	8
1445		1.6	-0.1	0.3	0.5	C	30	10
1447		11.5	1.3	2.1	2.3	C	25	13
1448		7.2	1.2	9.8	3.0	F	51	
1453		0.1	-0.1	0.4	1.3	C	36	6
1454		1.0	0.2	0.1	0.0	C	28	11
1457		6.0	3.0	3.7	2.5	F	53	
1459		1.1	-0.4	0.5	-1.9	C	41	2
1461		0.9	-0.5	0.2	-0.9	C	31	9
1461		2.0	-3.4	1.8	-7.0	F	52	
1470		2.5	1.2	1.0	0.9	C	27	12

1476		1.3	-0.6	0.3	0.6	C	30	11
1479		6.2	7.5	2.4	4.0	F	55	
1480		2.7	0.5	0.6	0.8	C	32	9
1481		3.0	4.0	1.3	2.6	C	45	1
1483		2.3	-1.2	0.1	0.0	C	38	6
1483		19.4	-10.4	0.9	-1.5	F	54	
1491		1.3	0.7	0.3	-0.7	C	28	12
1503		0.8	0.2	0.1	0.2	C	31	11
1598		4.3	0.8	0.1	-0.1	O	18	
1645		0.9	-0.3	0.5	0.1	C	48	4
1679		6.2	3.0	28.9	1.1	F	56	
1684		0.3	0.3	1.2	-0.1	O	20	
1688		0.6	-0.2	2.0	0.1	C	22	17
1690		0.1	0.0	3.1	0.0	C	21	18
1760		1.6	-0.4	0.0	0.0	C	25	18
2650		0.2	0.1	1.6	-0.7	C	44	42
2664		0.2	0.2	0.8	-0.7	O	43	
2676		1.1	0.3	0.8	0.0	C	44	43
2683	2658	1.6	0.0	1.5	0.4	O	44	
2735	2725	7.2	-0.8	18.0	0.4	O	46	
2737		1.8	-0.2	6.0	-0.2	O	45	
2740		2.8	0.3	2.7	1.4	C	47	45
2772	2754	8.5	2.7	17.5	-2.5	O	47	
2812		4.1	-2.0	3.7	1.7	C	48	46
2815		1.2	0.1	0.0	0.0	C	50	45
2820	2837	61.3	-2.0	89.9	24.1	F	57	
2824		2.2	1.1	1.9	0.0	C	51	46
2837		22.4	-1.7	24.2	0.1	O	48	
2849		0.2	1.1	0.1	0.0	C	52	47
2852		1.7	-1.2	0.1	0.1	C	53	47
2855		3.3	-43.0	98.1	2.6	F	59	
2856		89.1	100.0	52.7	-69.3	F	59	
2865		23.9	-22.0	19.0	4.6	F	60	
2866		14.9	-8.4	23.6	14.3	C	51	48
2870		0.6	2.4	0.3	0.2	C	54	47
2871		0.8	-3.7	0.7	0.3	C	55	47
2880		1.9	2.7	0.4	0.8	C	50	49
2882		44.7	-8.2	40.7	-17.7	F	65	
2886		25.7	-56.2	66.8	0.9	O	48	
2886		2.2	3.9	1.2	-4.7	C	51	49
2888		5.4	14.4	14.1	-32.0	O	49	
2891		1.9	2.3	5.0	0.2	C	51	50
2891		30.2	93.8	51.8	-15.6	C	52	50
2893		17.5	5.6	22.7	11.1	C	52	50
2897		7.8	-1.2	4.4	6.6	C	53	49
2899		85.4	-6.2	29.2	-14.2	F	63	
2899		0.2	0.1	1.2	0.2	C	56	38
2901		89.3	-34.1	100.0	-27.8	F	63	
2903		7.1	-2.6	9.4	-0.9	C	52	51
2904		34.3	30.3	74.8	22.2	F	64	
2907		42.6	-21.7	38.5	-11.2	C	52	49
2908		5.9	12.2	1.7	0.2	C	53	51
2909		1.0	1.4	0.9	1.5	C	56	39
2909		25.1	35.9	7.1	4.3	C	53	50
2910		28.7	-9.5	44.3	6.2	F	62	
2914		16.9	-53.3	23.3	54.4	C	54	49
2915		37.1	-32.1	52.5	-27.3	O	51	
2916		0.3	0.0	1.2	-0.1	C	55	48
2917		38.8	36.1	30.1	0.6	F	67	
2918		8.0	-25.7	2.3	4.5	C	54	49
2919		9.1	-9.5	11.5	22.1	C	53	52
2922		11.9	41.6	19.3	11.8	C	55	49
2924		1.8	-12.3	13.8	4.3	C	55	50
2926		0.1	-0.7	1.4	-0.8	C	54	51
2928		3.9	-23.5	3.2	-17.1	C	55	51
2928		10.9	-10.0	28.8	-6.2	C	56	40
2931		2.2	9.0	40.8	-31.4	O	53	
2931		5.6	-35.5	30.3	16.2	O	53	
2934		1.3	-3.2	4.7	15.8	C	54	52
2942		4.6	57.1	27.9	-79.3	C	55	52
2945		25.5	-34.4	17.1	23.6	C	55	53
2947		17.5	-63.9	43.5	13.6	C	55	54
2949		0.8	1.5	0.3	-0.7	C	56	41
2951		48.0	71.2	60.6	6.4	F	68	
2951		24.4	-21.9	14.2	-29.7	O	54	
2957		86.2	89.2	28.0	100.0	F	71	
2966		7.5	13.9	11.2	19.6	F	69	
2971		8.5	-16.0	7.9	-4.7	O	55	
2973		23.4	-0.5	52.5	-6.4	F	72	
2985		9.0	-6.5	40.6	-13.0	F	70	
2998		100.0	-18.3	87.8	-5.3	F	71	
3004		5.8	0.9	39.4	9.8	C	56	42
3019		4.5	0.5	7.9	1.0	C	56	43
3034		9.4	0.2	31.4	0.1	C	56	44
3064		0.4	0.0	1.5	0.0	C	56	46
3096		1.0	0.0	1.6	0.2	C	62	4
3101	3106	1.1	-0.1	1.7	0.6	C	66	3
3142	3141	1.1	0.1	1.6	0.2	C	67	5
3144		0.4	-0.1	1.2	-0.5	C	68	4
3196	3216	0.6	-0.2	0.7	-0.4	C	59	8
3349	3307	0.2	0.0	1.7	0.0	O	56	
3776	3768	0.1	0.0	1.0	0.0	C	72	18
4218	4234	1.1	-0.1	2.1	0.0	C	58	46
4262	4297	1.0	-0.2	1.2	-0.1	C	57	49
5570		1.7	0.1	0.7	-0.2	O	57	
5620		0.9	-0.2	0.3	0.1	O	58	
5850		0.7	0.1	0.1	0.0	O	72	

Table S5.: Camphor. Calculated transition frequencies (ν, cm^{-1}), dipole and rotational strengths, Raman and ROA intensities (absolute values of *D*, *R*, *Ram*, and *ROA*, in % of the maximal value), type of transition (F-fundamental, O-overtone, C-combination), and involved modes. Strongest 200 transitions are listed.

ν	ν_{exp}	*D*	*R*	*Ram*	*ROA*			
113		70.6	-17.0	0.4	-0.6	F	1	
155		1.2	13.2	0.1	0.1	F	2	
186		1.4	-7.3	0.0	-0.6	F	3	
216		2.3	-10.3	0.0	0.0	F	5	
220		1.8	-4.2	0.1	0.1	F	4	
230		0.2	-1.2	0.0	0.0	O	1	
243		6.8	-1.5	0.2	-0.8	F	6	
264		4.6	7.9	0.4	0.1	F	7	

286		5.0	-14.0	0.1	0.2	F	8	
300		9.1	5.1	0.3	-1.5	F	9	
382		3.4	4.9	0.2	1.0	F	10	
387		0.7	1.5	0.6	1.2	F	11	
416		4.3	7.3	0.1	-1.4	F	12	
475		2.6	6.7	0.8	4.6	F	13	
521		15.5	16.9	0.2	-2.0	F	14	
529		2.6	0.9	0.0	-0.3	C	12	1
549		1.3	-3.6	0.2	0.0	C	8	7
553		8.5	-50.8	1.9	0.8	F	15	
571		2.0	10.3	0.6	-0.5	F	16	
573		0.6	3.4	0.2	0.0	C	12	2
589		0.2	-1.2	0.0	0.1	C	13	1
604		3.3	-100.0	0.6	-1.7	F	17	
649		4.7	-15.7	11.0	1.8	F	18	
684		0.0	-1.3	0.1	-0.2	C	16	1
706		1.1	-18.0	1.7	-8.3	F	19	
719		0.2	-1.6	0.0	-0.4	C	17	1
731		0.4	1.1	0.0	0.0	C	16	2
748		23.2	86.4	0.7	-0.3	F	20	
822		0.2	-2.0	0.0	0.2	C	17	4
827		0.9	-6.5	0.3	2.2	F	21	
851		1.9	-46.6	1.1	5.5	F	22	
864		2.8	-33.7	3.2	-1.3	F	23	
871		0.1	-1.3	0.0	-0.1	C	16	9
906		0.5	-10.7	0.3	-1.0	C	20	2
910		1.8	-35.5	1.7	-7.3	F	24	
919		5.8	74.2	0.9	-2.5	F	25	
934		5.3	33.7	0.9	-7.5	F	26	
942		0.0	1.2	0.0	-0.1	C	15	11
943		0.2	-17.8	0.6	-0.1	F	27	
948		0.0	-1.0	0.0	0.1	C	19	6
949		0.3	-3.3	0.0	0.0	C	18	9
950		0.5	-6.3	0.2	0.4	O	13	
951		6.3	-5.2	2.7	6.4	F	28	
960		0.1	-1.1	0.0	0.0	C	16	11
984		0.6	-10.1	0.8	-2.8	F	29	
1009		1.1	15.0	0.8	1.4	F	30	
1017		19.9	-20.4	0.7	-0.8	F	31	
1018		3.0	-11.8	0.4	0.3	C	17	12
1023		0.7	-1.9	0.0	0.0	C	24	1
1031		0.7	-1.6	0.0	0.0	C	18	10
1034		0.3	-1.4	0.0	0.1	C	20	8
1037		44.5	-76.1	0.4	1.0	F	32	
1042		1.2	-1.9	0.0	0.0	C	21	5
1045		1.0	-0.8	0.0	0.1	O	14	
1048		9.1	-14.4	0.1	0.4	C	20	9
1075		3.3	-3.5	0.8	6.0	F	33	
1081		0.1	-0.9	0.0	0.2	C	23	4
1089		7.1	-2.3	1.7	-4.1	F	34	
1095		0.2	-2.8	0.1	0.2	C	26	2
1118		1.2	18.0	0.3	1.8	F	35	
1122		0.2	1.7	0.0	0.3	C	26	3
1125		0.5	4.8	0.0	0.7	C	16	15
1129		0.4	1.2	0.1	-0.1	C	17	14
1131		0.1	2.1	0.0	0.0	C	31	1

1145		0.6	-10.8	1.8	-6.1	F	36	
1149		0.3	-1.6	0.1	-1.2	C	26	5
1158		0.3	0.9	0.0	-1.0	C	27	5
1159		0.4	3.9	0.1	-0.8	C	27	4
1163		3.8	34.5	0.6	-4.7	F	37	
1167		1.4	12.8	0.2	-0.4	C	28	5
1168		0.1	1.3	0.0	0.0	C	28	4
1186		1.2	-13.0	1.1	9.1	F	38	
1195		3.4	-11.3	0.7	3.4	F	39	
1197		2.0	-4.7	0.4	1.9	C	24	8
1215		2.5	3.2	2.1	-0.5	F	40	
1234		0.3	3.4	0.1	-0.4	C	33	2
1235		0.1	2.7	0.1	0.1	C	35	1
1238		0.3	11.7	0.6	0.0	F	41	
1239		2.6	43.1	2.2	-10.8	F	42	
1245		0.2	3.6	0.2	-0.7	C	23	10
1249		0.1	1.1	0.1	-0.2	C	34	2
1250		0.0	0.9	0.1	-0.5	C	28	9
1255		0.6	4.5	0.1	-0.5	C	18	17
1266		2.1	3.9	0.3	-2.7	C	22	12
1266		5.2	-8.7	0.7	-3.5	F	43	
1272		4.3	2.0	0.6	-3.8	C	20	14
1277		0.2	1.4	0.1	-0.2	C	19	16
1278		0.9	-1.1	0.1	-0.6	C	23	12
1293		2.8	-16.7	2.4	-5.8	F	44	
1298		2.0	-4.8	1.0	2.0	F	45	
1299		0.5	1.0	0.3	0.6	O	18	
1311		0.2	-0.9	0.1	0.2	C	19	17
1321		10.6	30.7	3.0	-3.8	F	46	
1363		7.6	-0.8	0.1	0.3	F	47	
1371		4.3	6.4	0.4	0.2	F	48	
1384		16.0	-22.0	0.5	-0.4	F	49	
1396		12.3	-5.9	2.9	-9.3	F	50	
1427		0.8	-1.1	1.0	-0.7	C	43	2
1432		2.1	-1.9	1.4	-2.6	F	51	
1436		8.0	-7.2	2.8	12.8	F	52	
1440		4.7	17.8	1.7	-1.5	F	53	
1444		0.4	1.8	0.2	1.3	C	36	9
1446		3.1	4.1	0.6	6.4	F	54	
1448		6.2	1.8	0.7	3.2	F	55	
1454		0.1	1.0	0.0	0.3	C	20	19
1458		0.8	2.2	0.4	-2.2	C	26	14
1459		0.5	2.7	0.2	-0.6	C	29	13
1460		0.4	-0.4	1.5	-3.3	F	56	
1461		0.1	0.1	0.1	-1.1	C	39	7
1465		0.7	1.5	0.1	0.2	C	27	14
1466		5.6	19.1	0.7	-2.6	F	57	
1469		1.8	-2.7	1.1	1.9	F	58	
1474		0.9	1.7	0.1	-0.1	C	28	14
1476		1.2	-1.1	0.9	4.7	F	58	
1497		0.4	-2.0	0.1	0.3	O	20	
1680		0.6	1.1	0.0	-0.2	C	33	17
1727		1.1	0.7	0.0	-0.1	C	35	17
1739		2.1	-1.6	0.0	0.1	C	38	15
1743		11.9	6.7	0.4	-0.6	C	32	19
1744		1.0	1.3	0.0	-0.2	C	43	13

1750		2.0	0.0	0.1	0.1	C	39	15
1756		100.0	21.6	3.6	-3.6	C	38	16
1758		88.2	-10.2	3.3	-2.2	C	38	16
1760		11.2	4.4	0.4	-0.4	C	41	14
1761		44.0	-0.3	1.7	-1.4	C	24	22
1761		29.5	0.6	1.1	-0.7	C	26	21
1766		1.8	0.9	0.1	-0.4	C	31	20
1768		1.6	0.0	0.0	0.0	C	44	13
1768		1.0	0.0	0.0	-0.1	C	27	21
1768		4.7	-1.5	0.2	0.0	C	37	17
1769		1.5	-0.2	0.1	0.0	C	39	16
1771		1.5	-0.6	0.1	-0.1	C	25	22
1781		4.7	-0.5	0.1	-0.2	C	33	19
1788		7.5	2.1	0.3	-0.3	C	32	20
1790		1.3	1.3	0.1	-0.2	C	41	15
1793		1.5	1.5	0.1	0.1	C	36	18
1796		0.7	-1.5	0.0	0.2	C	46	13
1890	1899	0.7	-1.7	0.0	0.0	C	32	22
1958	1970	0.1	-1.1	0.0	0.1	C	32	25
2056	2066	0.2	1.5	0.0	-0.2	C	32	31
2113	2126	0.3	-2.5	0.0	0.0	C	33	32
2504	2509	0.0	0.0	0.1	-1.4	C	43	41
2717		0.0	0.0	1.6	0.0	O	47	
2726	2730	0.0	0.0	1.2	0.1	O	48	
2762		0.0	0.0	1.2	0.4	O	49	
2777	2774	0.2	-0.2	2.2	1.6	O	50	
2833		0.0	-1.1	0.0	0.0	C	55	49
2840		2.5	-1.0	26.7	-0.5	O	51	
2856		0.6	0.8	0.2	-1.3	C	56	50
2866		1.1	-4.8	1.3	-4.8	C	58	50
2866		0.8	1.8	0.8	-2.6	C	58	50
2867		0.6	1.0	0.4	-5.3	C	52	51
2867		1.7	3.0	2.9	-4.8	F	60	
2871		2.5	4.9	0.9	2.0	C	54	51
2879		0.2	-0.1	5.3	2.5	C	55	51
2881		1.5	1.1	4.5	1.1	C	54	52
2882	2872	0.1	1.4	1.6	-0.3	C	54	52
2884		0.9	4.8	1.5	2.7	C	55	51
2886		0.6	1.1	2.5	0.3	C	54	53
2888		2.4	2.2	5.3	-1.8	O	54	
2890		0.2	-1.7	0.4	1.9	C	55	53
2896		1.3	-1.0	1.6	5.1	C	56	52
2896		0.2	0.8	0.7	-1.2	C	55	54
2898		0.9	0.9	0.9	2.3	C	56	53
2899		0.1	-0.4	0.4	-1.6	C	56	53
2903		0.1	-0.1	0.7	0.9	C	58	51
2903		0.3	-1.1	0.5	-0.2	C	58	52
2906		0.1	0.0	0.4	-0.9	C	57	53
2908		3.3	6.9	3.8	3.2	C	56	55
2909		0.8	0.5	1.3	4.0	C	56	55
2911		0.5	1.1	4.0	1.0	C	58	53
2915	2872	0.6	0.5	15.3	0.8	O	56	
2919		0.4	-0.2	3.5	-2.5	C	58	55
2923		3.4	3.8	5.0	-2.5	C	58	54
2932		0.3	1.7	4.1	-1.9	O	57	
2935		0.6	2.2	1.1	1.3	C	58	57
2935		3.4	-1.8	47.7	66.3	F	64	
2940		10.6	-17.4	64.2	17.8	F	61	
2942		1.4	-4.4	31.0	-8.2	O	58	
2947		5.5	7.0	30.0	-16.5	F	60	
2951		14.7	-53.0	35.4	19.2	F	67	
2952		0.9	39.4	30.1	-35.2	F	66	
2956		4.1	77.6	39.3	-52.5	F	69	
2959		20.2	-98.0	59.3	77.1	F	66	
2960		0.5	1.1	100.0	-49.1	F	63	
2963		1.8	20.9	5.6	4.0	F	68	
2967		20.3	2.4	42.2	45.7	F	70	
2970		18.2	26.5	47.3	66.1	F	72	
2971		13.5	27.5	41.8	-1.6	F	71	
2974		10.9	-39.2	30.2	-91.7	F	73	
2975		11.6	39.1	76.0	-0.7	F	65	
2982		16.1	9.5	19.6	-100.0	F	74	
2995		10.0	-8.2	26.9	36.5	F	75	
2996		2.3	-4.8	3.3	11.4	C	59	41
3052		0.0	0.5	1.2	-6.9	C	59	44
3057		0.0	0.4	0.2	1.1	C	59	45
3122		0.1	0.1	0.9	-0.8	C	68	2
3157	3139	0.1	-0.8	0.9	0.2	C	71	3
3182		0.1	-0.3	0.9	0.2	C	70	5
3499	3468	1.7	-1.0	0.3	0.0	O	59	

Table S6.: Fenchone. Calculated transition frequencies (ν, cm^{-1}), dipole and rotational strengths, Raman and ROA intensities (absolute values of *D*, *R*, *Ram*, and *ROA*, in % of the maximal value), type of transition (F-fundamental, O-overtone, C-combination), and involved modes. Strongest 200 transitions are listed.

ν	ν_{exp}	*D*	*R*	*Ram*	*ROA*			
105		78.7	0.0	0.1	-0.2	F	1	
161		2.0	-5.9	0.0	0.1	F	3	
172		4.4	-4.0	0.0	-0.1	F	3	
193		12.5	-14.1	0.2	0.1	F	4	
206		20.7	-0.5	0.2	-0.5	F	5	
211		10.5	9.7	0.3	0.1	F	6	
255		7.9	-1.8	0.7	-0.6	F	7	
287		14.5	0.1	0.4	1.3	F	8	
291		53.0	4.8	0.3	0.2	F	8	
314		0.4	-1.2	0.1	-0.1	F	10	
350		7.0	-9.0	0.5	-0.4	F	11	
404		1.0	-2.0	0.1	0.1	C	6	5
417		3.9	4.7	0.5	-0.7	F	12	
421		0.5	-0.9	0.0	0.1	C	10	1
445		4.3	-5.6	0.5	0.6	F	13	
447		1.9	-3.0	0.2	0.3	C	11	1
505		7.8	2.3	0.4	0.0	F	14	
508		1.8	0.7	0.1	0.0	C	10	4
509		2.2	0.6	0.1	-0.1	O	7	
514		3.0	-1.6	0.1	0.0	C	10	5
517		6.5	-3.0	0.1	0.0	C	12	1
522		6.7	-2.9	0.2	0.0	C	12	1
596		2.8	0.7	5.4	-0.1	F	16	
655		10.1	-6.2	5.4	0.5	F	17	
713		1.0	-0.4	0.5	0.1	C	14	6

715		1.5	-0.8	1.0	0.2	F	18	
777		2.7	-3.4	0.5	-1.0	F	19	
813		3.8	-0.3	0.3	-0.1	C	15	9
815		15.7	6.9	1.5	0.2	F	20	
825		19.1	-16.5	1.5	2.4	F	21	
830		2.0	1.1	0.2	-0.2	O	12	
831		1.2	4.0	0.2	-0.2	O	12	
835		0.9	2.6	0.1	-0.2	C	15	10
882		3.2	-12.4	0.8	-0.4	F	23	
883		1.1	-2.6	0.8	0.5	C	18	3
887		2.1	-4.5	1.4	-0.2	F	24	
889		0.6	-0.7	0.1	0.0	O	13	
921		10.9	14.0	1.8	0.2	C	18	6
922		17.5	19.7	2.2	-0.1	F	25	
937		33.8	-24.5	1.6	-0.1	F	26	
944		2.1	2.4	0.5	1.0	F	27	
950		14.5	6.7	2.0	-2.6	F	28	
952		1.1	1.6	0.1	-0.2	C	19	2
983		19.3	-4.1	0.8	0.5	F	29	
983		0.7	0.8	0.0	0.0	C	20	3
984		1.3	-0.5	0.0	0.0	C	19	6
989		13.3	28.8	0.5	1.9	F	30	
991		5.2	14.3	0.3	1.2	C	20	2
999		0.9	-1.5	0.0	0.0	C	22	3
1007		100.0	74.5	0.5	0.2	F	31	
1013		22.4	-25.5	0.1	-0.2	C	20	5
1015		23.2	-56.8	0.6	-0.9	F	32	
1053		0.3	-1.0	0.0	-0.1	C	23	3
1059		0.4	-0.9	0.0	-0.3	C	23	2
1061		3.6	-0.6	0.7	-3.6	F	33	
1062		0.2	0.8	0.0	-0.2	C	24	2
1084		0.4	-0.9	0.0	-0.1	C	23	5
1096		22.8	-17.5	1.9	1.6	F	34	
1108		5.1	-7.0	2.3	-0.4	F	35	
1145		4.6	-1.6	1.7	2.2	F	36	
1159		4.2	-24.3	1.6	4.2	F	37	
1165		0.2	-0.9	0.1	0.1	C	30	2
1188		0.9	-0.9	0.2	-0.3	F	38	
1200		0.5	1.4	0.2	-0.4	C	31	4
1205		3.9	6.9	1.4	-3.3	F	39	
1207		1.4	1.6	0.1	-0.4	C	31	5
1214		4.1	3.1	0.3	0.5	C	31	6
1217		2.8	-1.6	1.4	0.3	F	40	
1221		1.3	0.2	0.5	0.0	C	18	14
1233		3.1	2.4	1.7	-2.5	F	41	
1240		2.0	-2.2	2.4	-0.1	F	42	
1258		4.1	0.5	1.8	-6.1	F	43	
1263		2.0	-0.3	0.8	-2.2	C	31	7
1282		9.8	9.3	0.4	1.7	F	44	
1283		3.4	2.2	0.2	0.5	C	35	2
1304		0.3	-0.9	0.1	0.0	C	34	6
1306		3.5	-6.7	0.8	0.4	F	45	
1312		2.1	-4.8	0.6	-0.1	C	18	16
1328		4.2	11.5	1.4	0.0	F	46	
1333		0.2	1.6	0.2	0.0	C	21	14
1344		0.9	0.6	0.1	-0.1	C	21	15

1351		10.8	4.7	0.1	0.1	F	47	
1370		8.6	-0.2	0.4	0.0	F	48	
1375		15.5	4.2	0.3	0.3	F	49	
1376		7.6	2.0	0.2	0.1	C	33	10
1427		2.0	1.2	0.7	-0.3	C	43	3
1428		0.9	-1.7	0.9	-0.7	C	43	3
1429		1.8	-0.1	2.7	-1.6	F	50	
1436		3.6	-8.0	0.0	0.4	F	52	
1437		6.9	0.5	1.4	1.6	F	53	
1439		0.7	-0.8	0.2	0.3	C	42	5
1439		0.9	0.7	0.2	0.0	C	25	15
1442		5.6	0.4	0.9	-0.7	C	36	9
1443		1.7	0.2	0.6	0.1	C	38	7
1444		1.1	-3.7	2.1	-2.0	F	55	
1446		18.1	19.8	1.3	0.2	F	54	
1447		6.1	4.4	0.5	-1.3	C	42	6
1449		2.0	-1.4	0.9	-1.5	C	37	8
1452		8.0	-2.6	0.3	0.3	C	27	14
1452		12.5	9.1	0.3	-0.4	F	56	
1453		0.9	0.7	0.1	0.0	C	43	4
1454		1.4	-0.7	0.1	0.0	C	37	9
1454		6.1	-1.6	0.5	2.7	C	28	14
1458		2.1	1.3	0.4	1.2	C	32	13
1460		1.6	0.0	0.1	0.2	C	39	7
1461		5.8	6.1	0.3	1.0	C	32	13
1469		2.8	-2.0	0.7	0.6	C	20	17
1473		9.4	-0.5	2.7	0.2	F	58	
1485		0.7	0.4	0.2	0.2	C	22	17
1491		1.0	0.4	0.1	0.0	C	19	18
1553		1.3	0.3	0.0	0.0	O	19	
1592		1.0	-0.8	0.1	0.0	C	20	19
1649		0.8	0.0	0.0	0.0	O	21	
1660		0.9	0.2	0.0	0.0	O	22	
1717		2.1	0.3	0.0	0.0	C	24	22
1722		3.5	2.0	0.1	-0.1	C	31	18
1724		1.6	0.1	0.0	0.0	C	39	15
1728		5.9	0.8	0.1	-0.1	C	32	18
1735		4.0	0.4	0.1	0.0	C	25	20
1742		9.0	0.8	0.1	0.0	C	46	12
1742		0.8	-0.3	0.0	0.0	C	55	9
1745		16.1	1.4	0.2	0.0	C	42	14
1746		32.0	-1.4	0.5	0.1	C	25	21
1750		49.7	-0.8	0.7	0.1	C	25	22
1752		2.0	0.6	0.0	0.0	C	45	13
1752		1.9	0.5	0.0	0.0	C	41	15
1754		1.4	1.0	0.0	0.0	C	37	16
1757		70.5	2.2	1.0	-0.1	C	42	15
1759		93.5	3.7	1.4	-0.2	C	27	20
1759		23.7	1.0	0.3	-0.1	C	27	20
1760		64.8	-4.9	1.0	0.3	C	29	19
1762		3.5	1.0	0.0	0.0	C	35	17
1762		46.2	-3.5	0.7	-0.2	C	26	21
1765		3.3	0.0	0.1	0.0	C	43	14
1767		11.2	-0.1	0.2	-0.1	C	30	19
1768		55.1	-1.0	0.9	-0.3	C	26	22
1770		1.5	0.0	0.0	0.0	C	27	21

1771		1.0	0.1	0.0	0.1	C	46	13
1772		3.9	0.7	0.1	0.0	O	24	
1775		1.0	-0.1	0.0	0.0	C	28	21
1778		3.4	0.3	0.1	-0.1	C	43	15
1791	1800	1.2	-0.9	0.0	0.1	C	32	19
1807		2.5	-0.1	0.0	0.0	C	25	24
1808		0.7	0.2	0.0	0.0	C	29	21
1812	1831	1.4	0.1	0.0	0.0	C	40	16
1833	1840	1.6	0.2	0.0	0.0	C	31	21
1841	1857	1.3	0.8	0.0	0.0	O	25	
1929	1923	1.3	0.0	0.1	0.0	C	31	25
2695	2711	0.0	0.0	1.1	0.0	O	47	
2723	2733	0.1	0.0	1.5	0.0	O	48	
2743	2758	0.0	0.0	1.1	0.0	O	49	
2844	2869	4.2	-0.4	24.8	0.1	O	52	
2845		2.0	-0.9	24.9	1.2	O	50	
2856		5.0	0.2	2.3	0.2	C	55	50
2858		0.0	-0.2	1.0	-0.5	O	51	
2863		4.4	2.8	5.8	-0.1	C	53	52
2873	2882	4.2	-6.7	2.2	-3.5	C	53	52
2876		4.3	-1.2	1.8	-1.9	C	57	50
2877		2.5	1.1	4.2	0.5	O	53	
2882		0.8	-0.1	11.3	-0.2	C	57	51
2886	2869	2.9	-2.3	14.9	2.6	C	56	52
2888		1.4	-1.2	0.6	-0.2	C	57	50
2888		0.3	0.5	11.1	-0.5	C	56	52
2889		1.2	-0.7	0.9	0.0	C	56	53
2890		0.2	0.2	1.1	0.1	C	55	54
2891		3.0	0.4	5.2	-1.1	C	55	54
2892		0.5	0.1	3.8	-0.3	O	54	
2896		1.3	-1.0	0.5	0.4	C	56	54
2900		0.7	0.1	4.4	0.1	C	57	55

2903		1.6	0.2	4.6	0.6	C	57	54
2906		1.5	0.0	0.1	0.5	C	57	56
2909		0.9	2.0	1.1	-1.3	C	58	53
2910		2.1	-0.9	1.2	-1.6	C	57	56
2912		4.8	-3.2	4.8	0.7	C	58	56
2914		1.1	1.3	0.7	-0.2	C	58	54
2915		1.7	-0.1	2.2	0.4	C	58	55
2921		2.5	0.1	1.1	-1.2	C	58	57
2938		10.1	-5.6	87.9	8.9	O	58	
2939		17.4	3.0	83.2	-1.6	F	60	
2944		12.7	2.7	100.0	-16.4	F	62	
2945		6.7	-6.8	6.3	11.5	F	61	
2947		3.7	0.6	53.4	3.3	F	63	
2950		22.2	-8.0	23.2	47.3	F	67	
2959		20.8	-16.1	83.9	-60.5	F	66	
2962		22.7	-9.7	99.1	-27.4	F	64	
2970		12.5	-9.3	22.6	-24.8	F	68	
2971		36.1	85.1	65.2	-9.3	F	73	
2972		21.3	24.5	23.8	-38.4	F	69	
2973		21.6	-100.0	31.0	-10.8	F	70	
2974		28.1	97.8	32.2	100.0	F	71	
2976		28.3	-91.0	29.8	1.5	F	72	
2979		23.8	10.6	67.1	58.4	F	73	
2985		32.6	27.0	49.7	-39.2	F	75	
2994		27.7	-0.8	28.4	-20.6	F	74	
3037		0.5	0.2	0.8	-1.2	C	64	1
3140	3151	0.1	-0.5	0.5	-0.9	C	69	3
3171	3168	0.1	0.0	0.5	0.8	C	70	5
3182		0.2	0.0	0.6	-1.0	C	72	6
3495	3462	2.8	-0.5	0.4	0.0	O	59	
4298	4299	0.3	0.1	0.8	0.0	C	60	48

Gaussian input examples - camphor

Default PCM

```
chk=cam.chk
%mem=10GB
%nproc=8
#b3lyp/6-311++G** nosymm freq=(roa,vcd,anharmonic)
scrf=(solvent=methanol)

camphor

 0 1
  6   -0.595811    1.778591   -0.858856
  6    0.050740    0.595798   -1.640518
  6    0.293139   -0.505003   -0.553441
  6    1.451357    0.075044    0.269906
  6    0.875670    1.257794    1.061342
  6   -0.595487    1.264844    0.599624
  1   -0.624814    0.197358   -2.420212
  1    0.974844    0.871387   -2.137912
  1   -0.031462    2.698243   -0.957195
  1   -1.626181    1.990870   -1.219188
  1    0.997730    1.073237    2.131118
  1    1.420143    2.177335    0.829174
  1   -1.272780    1.802303    1.268078
  6   -0.909284   -0.259651    0.431110
  6    0.543869   -1.899145   -1.095469
  1   -0.292680   -2.239696   -1.711357
  1    1.448505   -1.911469   -1.711895
  1    0.694780   -2.619973   -0.290828
  6   -2.291572   -0.547937   -0.168291
  1   -3.057748   -0.224133    0.497897
  1   -2.478811    0.014155   -1.232133
  1   -2.424598   -1.580820   -0.332280
  6   -0.805837   -1.068171    1.736727
  1   -1.545656   -0.715362    2.461977
  1   -1.013145   -2.122174    1.548705
  1    0.177948   -1.014325    2.212644
  8    2.595456   -0.310251    0.285301

  0.085645
```

United cavity

```
%chk=cam.chk
%mem=10GB
%nproc=8
#b3lyp/6-311++G** nosymm freq=(roa,vcd,anharmonic)
scrf=(solvent=methanol,read)

camphor

 0 1
  6   -0.595811    1.778591   -0.858856
  6    0.050740    0.595798   -1.640518
  6    0.293139   -0.505003   -0.553441
  6    1.451357    0.075044    0.269906
  6    0.875670    1.257794    1.061342
  6   -0.595487    1.264844    0.599624
  1   -0.624814    0.197358   -2.420212
  1    0.974844    0.871387   -2.137912
  1   -0.031462    2.698243   -0.957195
  1   -1.626181    1.990870   -1.219188
  1    0.997730    1.073237    2.131118
  1    1.420143    2.177335    0.829174
  1   -1.272780    1.802303    1.268078
  6   -0.909284   -0.259651    0.431110
  6    0.543869   -1.899145   -1.095469
  1   -0.292680   -2.239696   -1.711357
  1    1.448505   -1.911469   -1.711895
  1    0.694780   -2.619973   -0.290828
  6   -2.291572   -0.547937   -0.168291
  1   -3.057748   -0.224133    0.497897
  1   -2.478811    0.014155   -1.232133
  1   -2.424598   -1.580820   -0.332280
  6   -0.805837   -1.068171    1.736727
  1   -1.545656   -0.715362    2.461977
  1   -1.013145   -2.122174    1.548705
  1    0.177948   -1.014325    2.212644
  8    2.595456   -0.310251    0.285301

  0.085645

 radii=UA0
```
